# Dimethyl itaconate ameliorates cognitive impairment induced by a high-fat diet via the gut-brain axis in mice

**DOI:** 10.1186/s40168-023-01471-8

**Published:** 2023-02-21

**Authors:** Wei Pan, Jinxiu Zhao, Jiacheng Wu, Daxiang Xu, Xianran Meng, Pengfei Jiang, Hongli Shi, Xing Ge, Xiaoying Yang, Minmin Hu, Peng Zhang, Renxian Tang, Nathan Nagaratnam, Kuiyang Zheng, Xu-Feng Huang, Yinghua Yu

**Affiliations:** 1 Jiangsu Key Laboratory of Immunity and Metabolism, Jiangsu International Laboratory of Immunity and Metabolism, Department of Pathogen Biology and Immunology, Xuzhou Medical University, Xuzhou, 221004 Jiangsu China; 2 The Second School of Clinical Medicine, Xuzhou Medical University, Xuzhou, 221004 Jiangsu China; 3 Illawarra Health and Medical Research Institute (IHMRI) and School of Medicine, University of Wollongong, Wollongong, NSW 2522 Australia

**Keywords:** Itaconate, Cognition, Obesity, Gut microbiome, Microglia, Gut-brain axis

## Abstract

**Background:**

Gut homeostasis, including intestinal immunity and microbiome, is essential for cognitive function via the gut-brain axis. This axis is altered in high-fat diet (HFD)-induced cognitive impairment and is closely associated with neurodegenerative diseases. Dimethyl itaconate (DI) is an itaconate derivative and has recently attracted extensive interest due to its anti-inflammatory effect. This study investigated whether intraperitoneal administration of DI improves the gut-brain axis and prevents cognitive deficits in HF diet-fed mice.

**Results:**

DI effectively attenuated HFD-induced cognitive decline in behavioral tests of object location, novel object recognition, and nesting building, concurrent with the improvement of hippocampal RNA transcription profiles of genes associated with cognition and synaptic plasticity. In agreement, DI reduced the damage of synaptic ultrastructure and deficit of proteins (BDNF, SYN, and PSD95), the microglial activation, and neuroinflammation in the HFD-fed mice. In the colon, DI significantly lowered macrophage infiltration and the expression of pro-inflammatory cytokines (TNF-α, IL-1β, IL-6) in mice on the HF diet, while upregulating the expression of immune homeostasis-related cytokines (IL-22, IL-23) and antimicrobial peptide Reg3γ. Moreover, DI alleviated HFD-induced gut barrier impairments, including elevation of colonic mucus thickness and expression of tight junction proteins (zonula occludens-1, occludin). Notably, HFD-induced microbiome alteration was improved by DI supplementation, characterized by the increase of propionate- and butyrate-producing bacteria. Correspondingly, DI increased the levels of propionate and butyrate in the serum of HFD mice. Intriguingly, fecal microbiome transplantation from DI-treated HF mice facilitated cognitive variables compared with HF mice, including higher cognitive indexes in behavior tests and optimization of hippocampal synaptic ultrastructure. These results highlight the gut microbiota is necessary for the effects of DI in improving cognitive impairment.

**Conclusions:**

The present study provides the first evidence that DI improves cognition and brain function with significant beneficial effects via the gut-brain axis, suggesting that DI may serve as a novel drug for treating obesity-associated neurodegenerative diseases.

Video Abstract

**Supplementary Information:**

The online version contains supplementary material available at 10.1186/s40168-023-01471-8.

## Introduction

Neurodegenerative diseases, such as Alzheimer’s disease (AD) and related dementias, have been recognized as a leading factor in morbidity and declined life quality in the aging population, which place a heavy burden on healthcare resources and severely hamper the development of the economy [1]. It has been reported that Western dietary patterns and obesity are risk factors for cognitive impairment and neurodegenerative diseases [2, 3]. For example, the epidemiological investigation showed that obese people who consume high-fat (HF) diets exhibit worse cognitive function [4]. Also, animal experiments supported that HF diet-induced obesity can impair hippocampus-dependent learning and memory [5–7]. Recently, accumulating research has shown that altering the gut-brain axis is involved in obesity-induced cognitive decline [5, 8]. For example, HF diet-induced gut microbiome alterations can induce cognitive impairment in mice [8]. In addition, obese-type microbiome transplantation has been shown to disrupt the intestinal barrier and induce a cognitive decline in mice [9].

The crosstalk between intestinal immune cells and microbiome is essential for gut homeostasis, and consequently regulating cognition [10, 11]. It is reported that intestinal immune cells and their derived cytokines affect gut microbiome composition [12]. For example, IL-22 promotes the secretion of antimicrobial peptides (e.g., Reg 3γ) in epithelial cells, thereby alleviating gut dysbiosis induced by pathogens [12]. Conversely, the absence of IL-22 or IL-23 drives gut microbiome shift in HF diet mice [13]. Furthermore, disturbed intestinal immunity and microbiome are associated with neuroinflammation, where damage to the epithelial barrier and intestinal mucus allows the entry of exterior antigens from the gut lumen into the host [14–16]. In obesity, intestinal permeability increases the hyper-translocation of bacterial lipopolysaccharide (LPS) into the blood circulation, allowing LPS to act as endotoxin on microglia and trigger neuroinflammation and cognitive impairment [8]. Hence, manipulating gut immune and microbiome homeostasis present an effective strategy for obesity-associated cognitive impairment.

Recently, itaconate has emerged as a novel negative regulator of the inflammatory response [17–19]. Itaconate can block pro-inflammatory responses in macrophages by inhibiting succinate dehydrogenase activity [17]. Moreover, itaconate suppresses the LPS-induced transcription of pro-inflammatory cytokines [18]. Furthermore, the derivatives of itaconate have beneficial effects in treating inflammatory diseases [20, 21]. For example, dimethyl itaconate (DI), a cell-permeable itaconate derivative, downregulates pro-inflammatory cytokines and reduces inflammation in a mouse model of fungal keratitis [21]. Intraperitoneal administration of DI can limit microglia activation and neuroinflammation in a mouse model of experimental autoimmune encephalomyelitis (EAE) [22]. However, it is still obscure whether DI can improve the alteration of gut immunity and microbiome, and consequently neuroinflammation and cognitive deficits induced by HF diet.

In the present study, a HF diet-fed mouse model with gut dysfunction and cognitive impairment was used to evaluate DI’s effects on improving the gut-brain axis. First, we assessed the effects of DI supplementation on colonic immune homeostasis, barrier function, and gut microbiome profile. Moreover, the pro-cognitive efficacy of DI concerning cognitive behavior, synaptic ultrastructure, and anti-neuroinflammation was investigated in the hippocampus, a region regulating cognitive function. In addition, RNA sequencing (RNA-seq) was performed to understand the effects of DI supplementation on cognition at the transcriptomic level. Furthermore, we utilized fecal microbiome transplantation (FMT) experiment to confirm the role of gut microbiome in the beneficial effects of DI supplementation on cognitive function. This study demonstrates for the first time that DI can prevent cognitive deficit via the gut-brain axis, which provides a novel point for treating obesity-related neurodegenerative diseases.

## Results

### DI supplementation improved cognitive decline in mice on HF diet

**Fig. 1:**
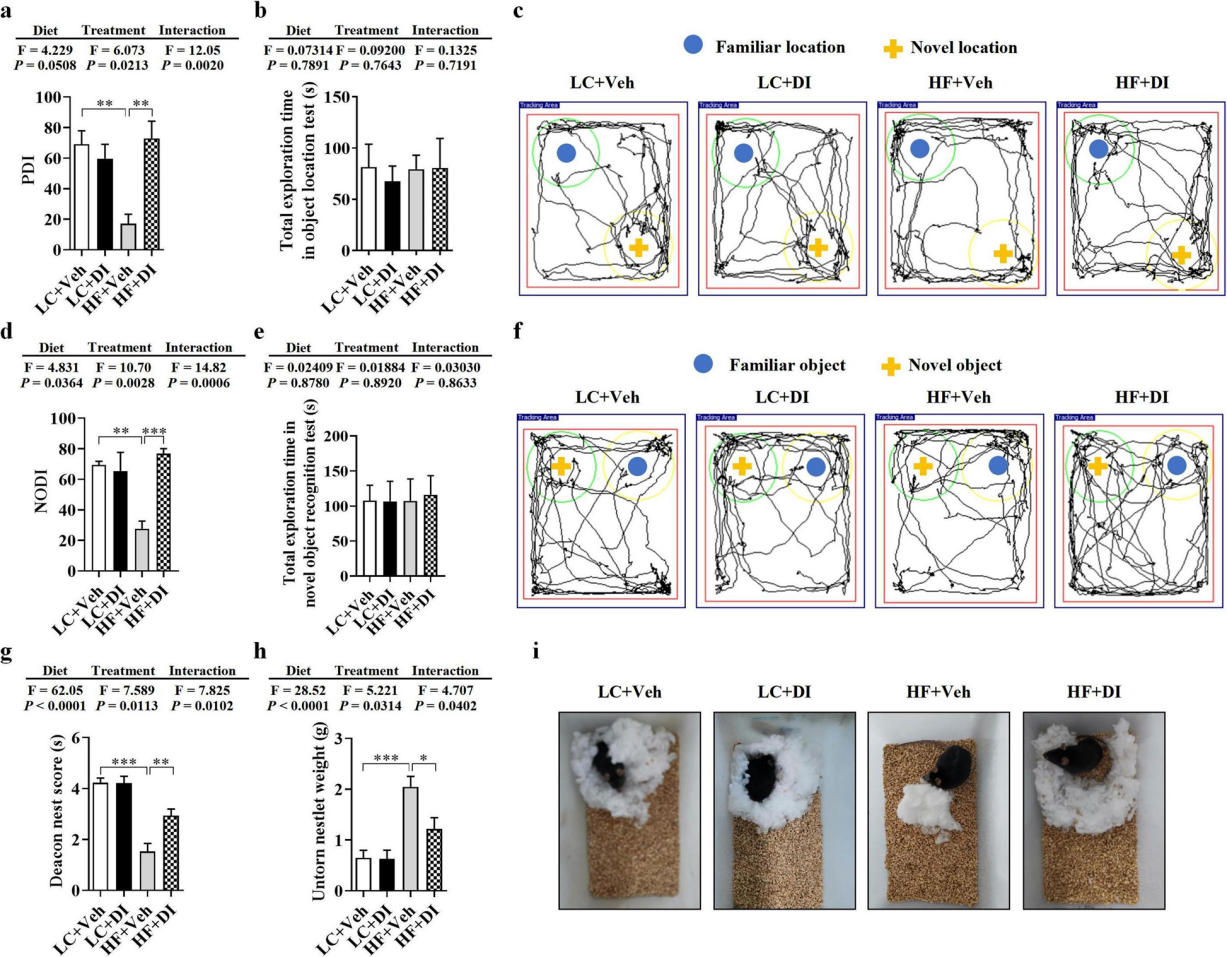
DI supplementation improved cognitive decline in mice on HF diet. The object location test was performed to evaluate the spatial memory of mice (**a**–**c**). **a** Percentage of time spent with the object in the novel place to total object exploration time. **b** The total object exploration time. **c** Representative track plots of LC+Veh, LC+DI, HF+Veh, and HF+DI groups recorded by the SMART video tracking system in the testing phase. Note that the LC+Veh mouse spent more time exploring the object in the novel place whereas the HF+Veh mouse did not show preference to the object in a novel place. The novel object recognition test was performed to evaluate the object recognition memory of mice (**d**–**f**). **d** Percentage of time spent with the novel object to total object exploration time. **e** The total object exploration time. **f** Representative track plots of LC+Veh, LC+DI, HF+Veh, and HF+DI groups recorded by the SMART video tracking system in the testing phase. The nest building test was used to assess the activities of daily living of mice (**g**–**i**). **g** The nest score and **h** untorn nestlet weight (amount of untorn nesting material). **i** Representative nest result of LC+Veh, LC+DI, HF+Veh, and HF+DI groups. *n* = 12 mice for each group in behavior tests. Values are mean ± SEM. ^*^P < 0.05, ^**^P < 0.01, ^***^P < 0.001

To investigate the effects of DI supplementation on cognitive decline induced by the HF diet, we performed object location, novel object recognition, and nesting behavioral tests, reflecting hippocampus-dependent recognition memory and ability to perform activities of daily living. In the object location test, place discrimination index (PDI), the percentage of time spent with the object in a novel place, was significantly decreased in mice on HF diet; however, DI supplementation significantly improved place recognition memory, increasing the PDI in mice on HF diet (*P* < 0.05, Fig. 1a, c). In the novel object recognition test, DI supplementation effectively prevented the decrease of the novel object discrimination index (NODI), percentage of time spent with a novel object, in mice on the HF diet (*P* < 0.05, Fig. 1d, f). The difference in PDI and NODI between the HF- and vehicle-treated mice and HF- and DI-treated mice was not due to general activity variation, as the total exploration time of the objects during the testing phase was comparable among the four groups (both *P* > 0.05, Fig. 1b, e). In the nesting behavior test, the ability to build a nest was impaired in mice on the HF diet with lower deacon nest score and higher untorn nestlet weight, while HF treated with DI exhibited a higher ability to build a nest (both *P* < 0.05, Fig. 1g–i). These results indicate that DI supplementation potently attenuates cognitive deficits caused by a chronic HF diet.

Furthermore, compared with the LC diet group, the mice on the HF diet increased their body weight, liver mass, and subcutaneous, epididymal, and brown fats (all *P* < 0.05, Fig. S1a-g). DI supplementation did not change the above indices of mice fed on either HF or LC diet (all *P* > 0.05, Fig. S1a-g). Also, there was no significant difference in energy intake of HF mice treated with DI compared with the HF group (*P* > 0.05, Fig. S1h). In addition, compared with LC diet-fed group, HF diet-fed mice showed insulin resistance and glucose intolerance, which were supported by increased blood insulin and glucose levels, homeostasis model assessment-insulin resistance (HOMA-IR) (*P* < 0.05, Fig. S1i-l). However, DI supplementation could not improve the insulin resistance (*P* > 0.05, Fig. S1i, j), and even worsen the glucose tolerance (*P* < 0.001, Fig. S1k, l) in HF diet-fed mice.

### DI supplementation improved the transcriptome profile associated with cognition in the hippocampus of mice on HF diet

**Fig. 2:**
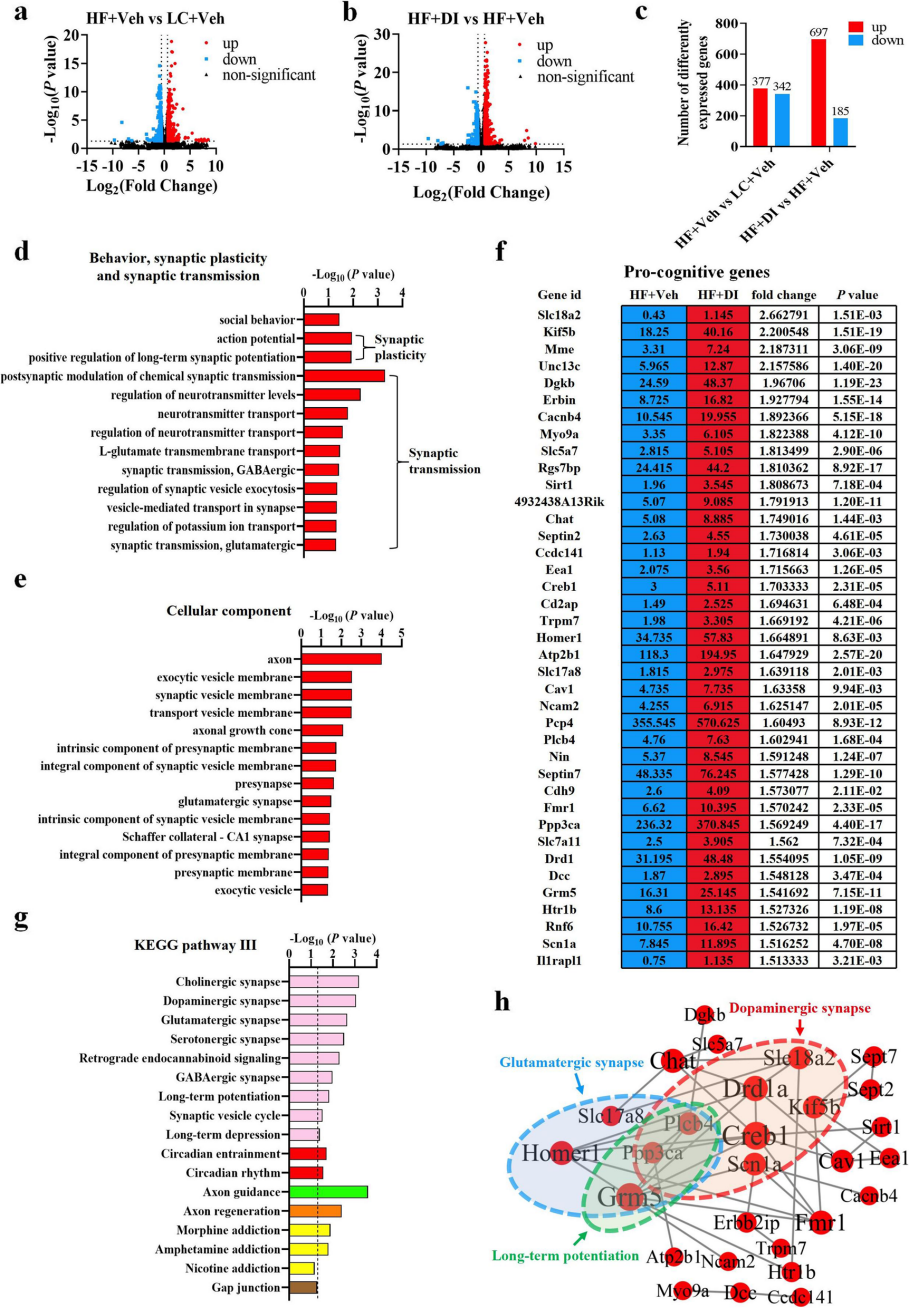
DI supplementation improved the transcriptome profile associated with cognition in the hippocampus of mice on HF diet. **a** The volcano plot shows the distributions of differentially expressed genes (DEGs) between HF+Veh and LC+Veh mice. **b** The volcano plot shows the distributions of DEGs between HF+DI and HF+Veh mice. **c** The number of upregulated and downregulated DEGs. **d** The biological processes associated with behavior, synaptic plasticity, and transmission are upregulated in HF diet-fed mice after DI supplementation. **e** The cellular components associated with synapse are upregulated in the HF+DI group in comparison to HF+Veh group. **f** The fpkm value, fold change and *P*-value of pro-cognitive DEGs of HF+Veh and HF+DI groups. **g** The enriched KEGG pathways related to behavior, synapse, and brain development in HF diet-fed mice after DI supplementation. Columns with different colors represent different classification in level 2. The dotted line in the figure represents *P* = 0.05. **h** PPI network analysis of pro-cognitive genes based on STRING database and Cytoscape 3.9.1. Black lines denoted the interaction between two proteins. *n* = 3 mice for each group

To further understand molecular mechanisms involved in DI preventing HF diet-induced cognitive impairment, we next performed RNA sequencing to reveal the global gene expression profile in the hippocampus, a key brain region regulating cognition. Differentially expressed genes (DEGs) were identified after filtering the raw data based on *P* < 0.05 and fold changes greater than 1.5 (Table S1-S4). There were 719 DEGs (including 377 upregulated genes and 342 downregulated genes) in the mice on the HF diet compared with the LC diet (Fig. 2a, c and Table S1, S2). There were 697 upregulated genes and 185 downregulated genes in mice on HF diet treated with DI compared with mice on HF treated with vehicle (Fig. 2b, c and Table S3, S4). Gene ontology (GO) analysis was carried out to seek the significantly enriched terms in upregulated DEGs in HF diet-fed mice with DI supplementation (Table S5, S6). The bubble chart showed the top 30 enriched GO terms of biological process and cellular components (Fig. S2a and b). Notably, significantly enriched terms were identified associated with behavior, synaptic plasticity, and synaptic transmission (Fig. 2d, e). In agreement, a glut of universally acknowledged pro-cognitive genes enriched in the above GO terms were significantly upregulated, including Slc18a2, Kif5b, and Mme (Fig. 2f). For example, Slc18a2 (also known as VMAT2) was reported to enhance dopamine release and protect against Parkinson’s disease-related neurotoxin insult in vivo [23], while its deficiency contributes to dopamine neuron death in the brain [24]. Moreover, KEGG analysis showed that the axon guidance and synapse-related pathways (long-term potentiation, dopaminergic synapse, glutamatergic synapse, etc*.*) were significantly enriched, ranked among the top 30 pathways (Table S7, Fig. S2c, Fig. 2g). In addition, we mapped the pro-cognitive genes measured above onto the protein-protein interactions (PPI) network (Fig. 2h). The PPI network indicated that Ppp3ca (also known as calcineurin) and Plcb4 (modulating chemical synaptic transmission [25, 26]) were two shared nodes among glutamatergic synapses, dopaminergic synapses, and long-term potentiation pathways (Fig. 2h). Overall, these results suggest that DI supplementation exceedingly alters the transcriptomic profile that links with cognitive function in the hippocampus of mice on the HF diet, supporting DI’s role in cognitive improvement.

### DI supplementation mitigated synaptic impairment and neuroinflammation in the hippocampus of mice on HF diet

**Fig. 3:**
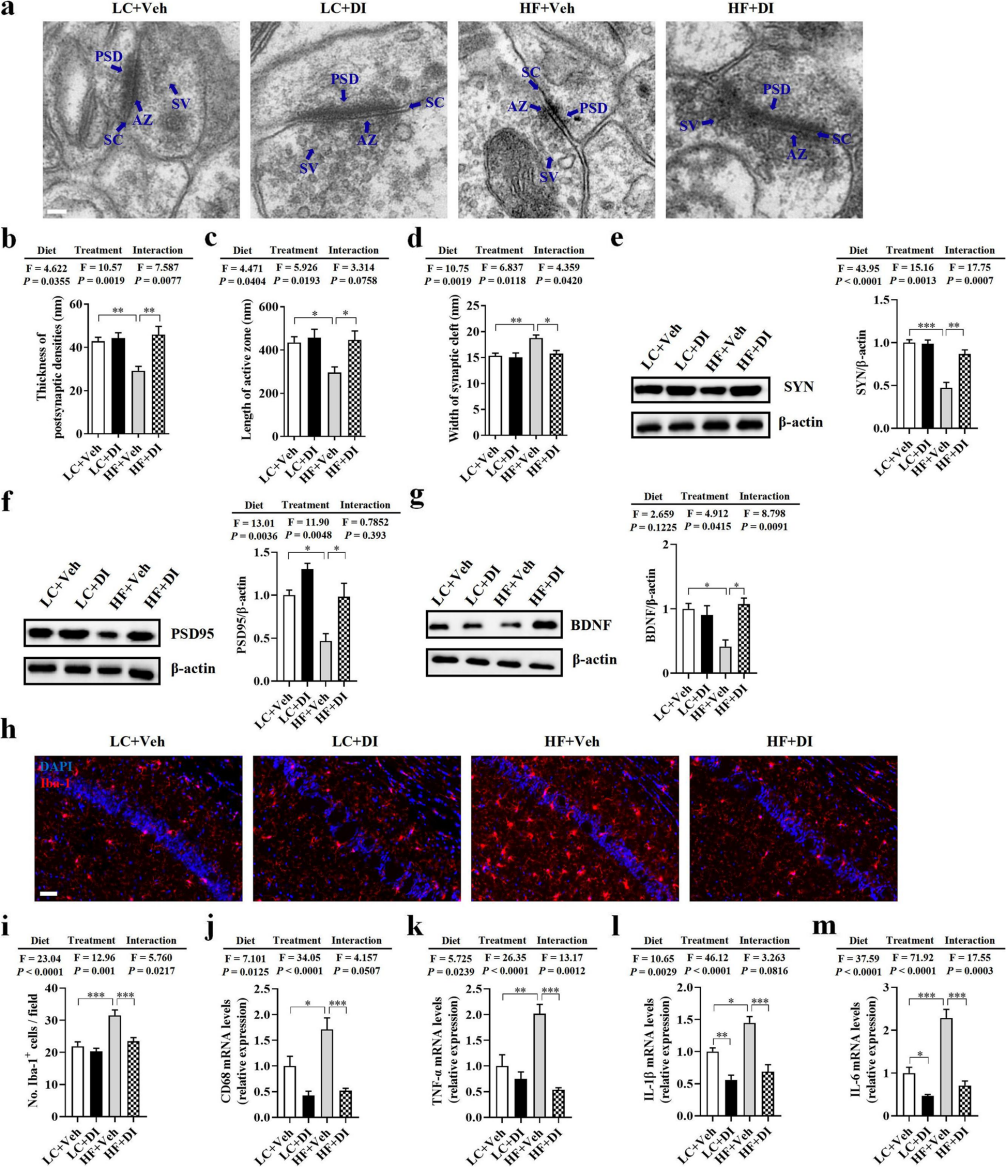
DI supplementation mitigated synaptic impairment and neuroinflammation in the hippocampus of mice on HF diet. **a** Representative ultrastructure of synapses in the cornu ammonis 1 (CA1) region of mice on the electron micrograph (scale bar: 100 nm). **b**–**d** Image analysis of the thickness of postsynaptic density (PSD), length of the active zone (AZ), and width of the synaptic cleft (SC) (*n* = 2, 8 images per mouse). **e**–**g** The protein expression levels of SYN, PSD95, and BDNF in the hippocampus (*n* = 5). **h** The immunofluorescent staining of Iba-1 in CA1 of the hippocampus. **i** The quantification of Iba-1^+^ cells in CA1 of the hippocampus (*n* = 3, 5 images per mouse, scale bar: 50 μm). **j–m** The mRNA expression of CD68, TNF-α, IL-1β, and IL-6 in the hippocampus (*n* = 6). Values are mean ± SEM. ^*^P < 0.05, ^**^P < 0.01, ^***^P < 0.001

Following our observation that DI supplementation improved cognitive decline, we further evaluated synaptic ultrastructure using transmission electron microscopy in the hippocampus’s cornu ammonis 1 (CA1) region. We observed that the HF diet decreased the thickness of the postsynaptic densities (PSD), shortened the length of the active zone (AZ), and broadened the synaptic cleft (SC) (all *P* < 0.05, Fig. 3a–d). However, compared with the HF+Veh group, DI supplementation attenuated these synaptic ultrastructure impairments, exhibiting thicker PSD, longer AZ, and narrower SC (all *P* < 0.05, Fig. 3a–d). Next, we measured the protein levels of synaptic plasticity markers (BDNF), and pre- and postsynaptic proteins (SYN and PSD95) in the hippocampus of mice. We found that DI supplementation significantly attenuated HF diet-induced expression downregulation of BDNF, SYN, and PSD95 at protein levels compared to the HF diet-fed mice (all *P* < 0.05, Fig. 3e–g). These results suggest that DI supplementation alleviates synaptic ultrastructure and protein deficits in HF diet-fed mice, contributing to cognitive improvement.

The activation of microglia in the hippocampus can impair the synaptic ultrastructure, which is an underlying mechanism to explain the pathogenesis of neurodegenerative diseases [27]. Using Iba-1 as the microglia marker, we observed that the HF diet increased microglia number in the CA1 of the hippocampus, while DI supplementation significantly reduced the cell number in this area (*P* < 0.05, Fig. 3h, i). Notably, mRNA expression of the activated microglial marker (CD68) was significantly higher in the hippocampus of the HF+Veh group than in the LC+Veh and HF+DI groups (*P* < 0.05, Fig. 3j). Moreover, the mRNA levels of pro-inflammatory cytokines (TNF-α, IL-1β, IL-6) were significantly higher in the hippocampus of the HF+Veh group than in the LC+Veh and HF+DI groups (all *P* < 0.05, Fig. 3k–m). These results show that DI supplementation attenuates neuroinflammation induced by a HF diet.

### DI supplementation restored colonic immune homeostasis and alleviated colonic mucosa barrier impairment in mice on HF diet

**Fig. 4:**
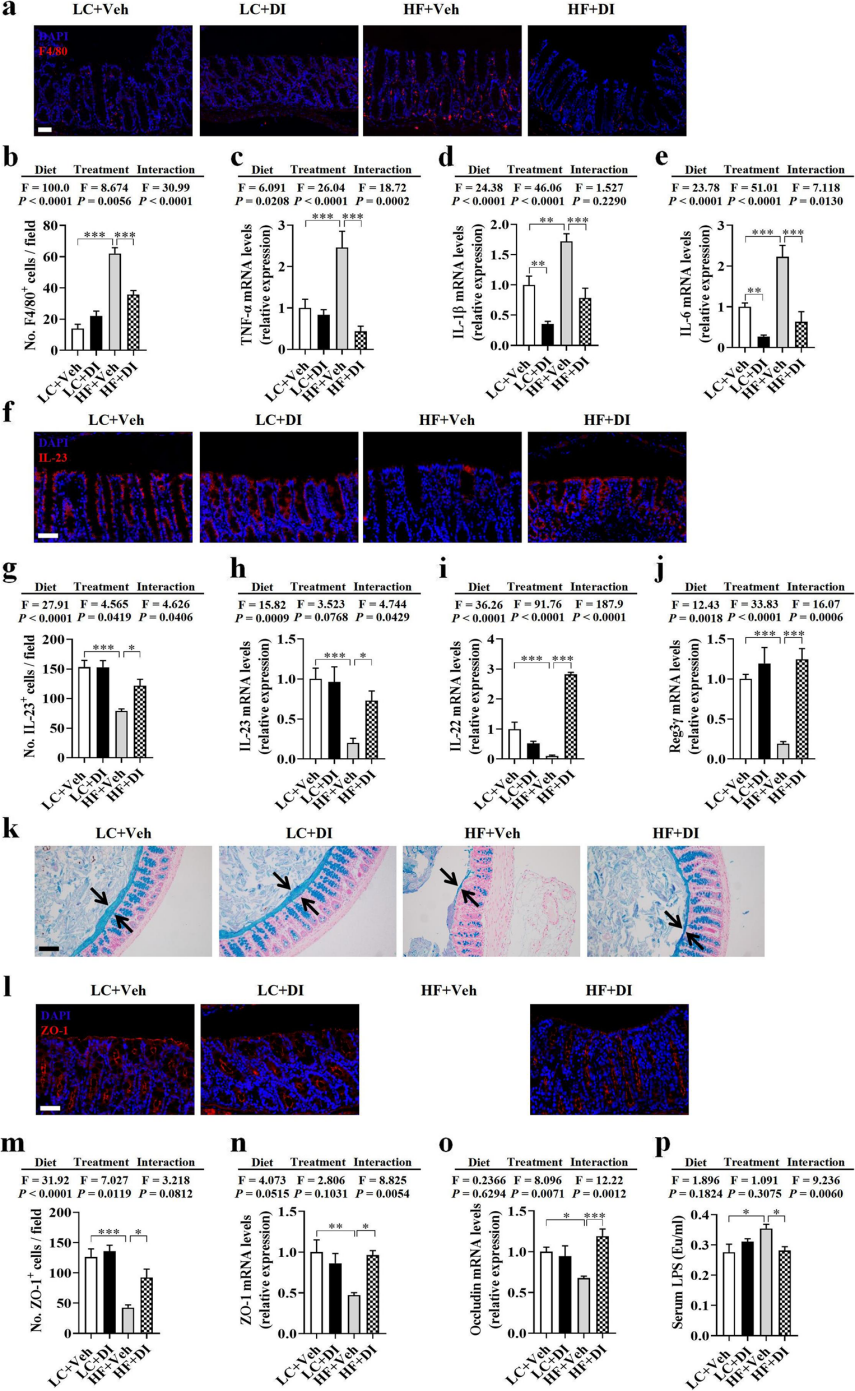
DI supplementation restored colonic immune homeostasis and alleviated colonic mucosa barrier impairment in mice on HF diet. **a** Immunofluorescence images of colonic sections stained with F4/80 (red) and DAPI (blue). **b** The quantification of F4/80^+^ cells *(n* = 3, 5 images per mouse, scale bar: 50 μm). **c**–**e** The mRNA expression of TNF-α, IL-1β, and IL-6 in the colon (*n* = 6). **f** Immunofluorescence images of colonic sections stained with IL-23 (red) and DAPI (blue). **g** The quantification of IL-23^+^ cells (*n* = 3, 5 images per mouse, scale bar: 50 μm). **h–j** The mRNA expression of IL-23, IL-22, and Reg3γ in the colon (*n* = 6). **k** Alcian blue-stained colonic sections were showing the mucus layer (arrows). (scale bar: 100 μm). Opposing black arrows with shafts delineate the mucus layer measured. **l** Immunofluorescence images of colonic sections stained with ZO-1 (red) and DAPI (blue). **m** The quantification of ZO-1^+^ cells (*n* = 3, 5 images per mouse, scale bar: 50 μm). **n, o** The mRNA expression of ZO-1, and occludin in the colonic tissues (*n* = 6). **p** The serum levels of LPS (*n* = 7). Values are mean ± SEM ^*^P < 0.05, ^**^P < 0.01, ^***^P < 0.001

Recently, it has been reported that DI suppresses the inflammatory response in cultured macrophages exposed to the endotoxin, LPS [17–19]. Here, we examined whether DI reduces colonic inflammation induced by the HF diet in vivo. Using F4/80 as a macrophage marker, we verified that the HF diet significantly increased the number of macrophages in the colon, which was decreased by DI supplementation (*P* < 0.05, Fig. 4a, b). Moreover, the mRNA levels of pro-inflammatory cytokines (TNF-α, IL-1β, IL-6) were significantly decreased in HF diet-fed mice after DI supplementation (all *P* < 0.05, Fig. 4c–e). Furthermore, DI supplementation increased the IL-23 level and mRNA expression in the colon of mice on the HF diet (all *P* < 0.05, Fig. 4f–h). It is known that IL-23 activate Th17 to produce cytokine IL-22 [28], which increases the secretion of antimicrobial peptides (e.g., Reg3γ) in the colon [11, 29]. We found that DI supplementation increased the mRNA expression of IL-22 and Reg3γ in the colon of mice on the HF diet (all *P* < 0.05, Fig. 4i, j). These results indicate that DI supplementation prevents colonic inflammation and restores intestinal immune homeostasis in mice on the HF diet.

The integrity of the mucosa barrier is closely associated with immune homeostasis [30]. Therefore, following the evidence that DI inhibited colonic inflammation, we further examined whether DI supplementation could improve colonic mucosa barrier impairment induced by HF diet feeding. To begin with, alcian blue staining showed that the reduction of mucus thickness by HF diet was reversed after DI supplementation (Fig. 4k). Moreover, DI supplementation elevated the zonula occludens-1 (ZO-1, a tight junction protein) level and mRNA expression in mice on the HF diet (all *P* < 0.05, Fig. 4l–n). In addition, DI supplementation upregulated the mRNA levels of occludin (another tight junction protein) in the colon of mice on the HF diet (*P* < 0.05, Fig. 4o). Furthermore, DI supplementation attenuated serum LPS levels elevated by the HF diet (*P* < 0.05, Fig. 4p), suggesting that DI enhanced gut barrier integrity and attenuated gut permeability. Thus, DI supplementation relieved the impairment of the colonic mucosa barrier in HF diet-fed mice.

### DI supplementation partially ameliorated gut microbiome shift in mice on HF diet

**Fig. 5:**
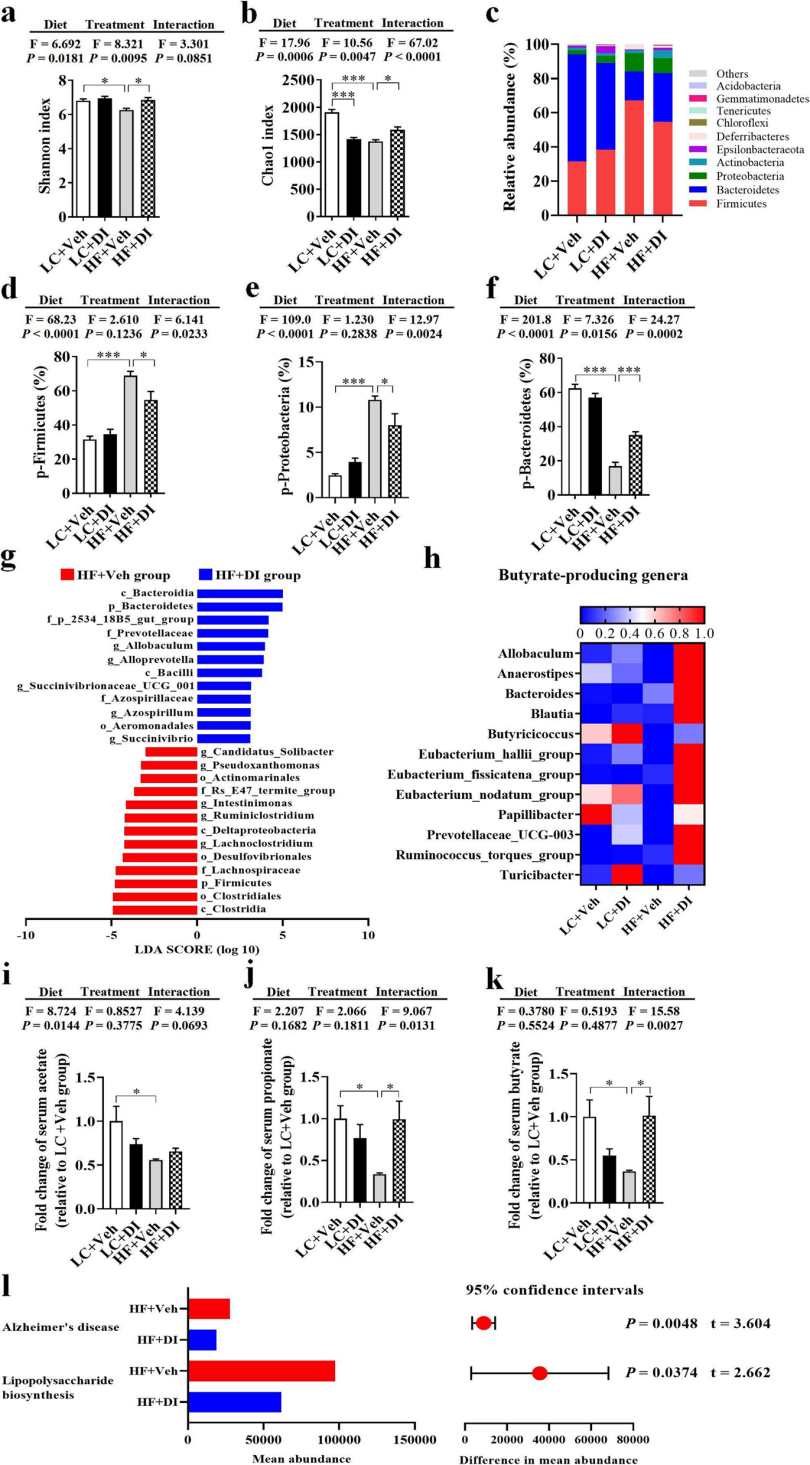
DI supplementation partially ameliorated gut microbiome alternation in mice on HF diet. Fecal microbiome composition was analyzed by 16S rRNA gene sequencing (*n* = 6). **a** Shannon index. **b** Chao1 index. **c** Composition of abundant bacterial phyla. **d–f** Relative abundance of Firmicutes, Proteobacteria, and Bacteroidetes. **g** Linear discriminant analysis (LDA) effect size (LEfSe) showing the most significantly abundant taxa enriched in microbiome from the HF+DI group compared to the HF+Veh group. **h** The heatmap of relative abundance of genera associated with the production of butyrate. The intensity of color in the heatmap (blue to red) indicates the normalized abundance score for each genus. **i**–**k** The relative serum levels of acetate, propionate, and butyrate (*n* = 6). **l** Predicted KEGG functional pathways associated with Alzheimer’s disease and lipopolysaccharide biosynthesis at level 3 inferred from 16S rRNA gene sequences using PICRUSt. Values are mean ± SEM. ^*^P < 0.05, ^**^P < 0.01, ^***^P < 0.001. Abbreviations: p, phylum; c, class; o, order; f, family; g, genus

Studies reported that disturbed microbiome can be reshaped by gut immune homeostasis-related factors (e.g., IL-22, IL-23, and Reg3γ) [11, 31]. Therefore, we investigated whether DI supplementation affects the diversity and composition of gut microbiome in mice on a HF diet. 16S rRNA sequencing showed that both diversity and abundance of bacteria became disordered after a chronic HF diet. Compared with the lab chow (LC) diet, the α-diversity, the Shannon indexes, and Chao1 indexes of the HF diet were significantly decreased, while DI supplementation significantly increased the two indexes of α-diversity (both *P* < 0.05, Fig. 5a, b). Moreover, DI supplementation reduced the abundance of Firmicutes and Proteobacteria (both *P* < 0.05, Fig. 5c–e) and increased Bacteroidetes (*P* < 0.05, Fig. 5c, f). Furthermore, DI supplementation mainly decreased the abundance of Clostridia at the class level, Clostridiales at the order level, Lachnospiraceae, and Ruminococcaceae at the family level, *Lachnospiraceae_NK4A136_group*, *Lachnoclostridium*, *Ruminiclostridium*, *Oscillibacter*, and *Intestinimonas* at the genus level in the Firmicutes (all *P* < 0.05, Fig. S3a). DI supplementation mainly decreased the abundance of Deltaproteobacteria at the class level, Desulfovibrionales at the order level, Desulfovibrionaceae at the family level, and *Desulfovibrio* at the genus level in the Proteobacteria (all *P* < 0.05, Fig. S3b). DI supplementation mainly increased Bacteroidia at the class level, Bacteroidales at the order level, Prevotellaceae at the family level, and *Alloprevotella* at the genus level in the Bacteroidetes (all *P* < 0.05, Fig. S3c). Intriguingly, DI supplementation also increased *Lactobacillus* (all *P* < 0.05, Fig. S3d). Overall, DI supplementation could partially rescue HF diet-induced alterations in gut microbiome composition.

Linear discriminant analysis (LDA) effect size (LEfSe) showed that DI supplementation decreased the abundance of Firmicutes, Proteobacteria, and their lower taxa and increased the abundance of Bacteroidetes and its lower taxa in HF diet-fed mice (LDA score > 3.0, Fig. 5g). Therefore, DI supplementation could partially rescued HF diet-induced alterations in gut microbiome composition. Intriguingly, we found that DI supplementation increased many genera associated with the production of butyrate (Fig. 5h). Moreover, the major propionate-producer, Bacteroidetes [32], was also significantly increased (*P* < 0.05, Fig. 5f). Then, we performed gas chromatography-mass spectrometry (GC-MS) to reveal the levels of short-chain fatty acids (SCFAs, including acetate, propionate, and butyrate) in the serum of mice. HF diet decreased the above SCFAs (all *P* < 0.05, Fig. 5i–k); however, DI supplementation increased serum propionate and butyrate in mice fed on HF diet (both *P* < 0.05, Fig. 5j, k). KEGG functional orthologs predicted by PICRUSt identified potential functional interactions between the gut microbiome and host among the four groups (Table S8-S10). Hierarchical clustering separated the HF with the vehicle group as a single cluster from the other three groups (Fig. S4a, b). Notably, the pathways associated with “Alzheimer’s disease” and “lipopolysaccharide biosynthesis” were significantly lower in the HF with DI group than those in the HF with the vehicle group (both *P* < 0.05, Fig. 5l). These results indicate that DI supplementation partially alleviates gut microbiome shift induced by the HF diet.

### DI supplementation improved cognition and synaptic ultrastructure through the gut microbiome

**Fig. 6:**
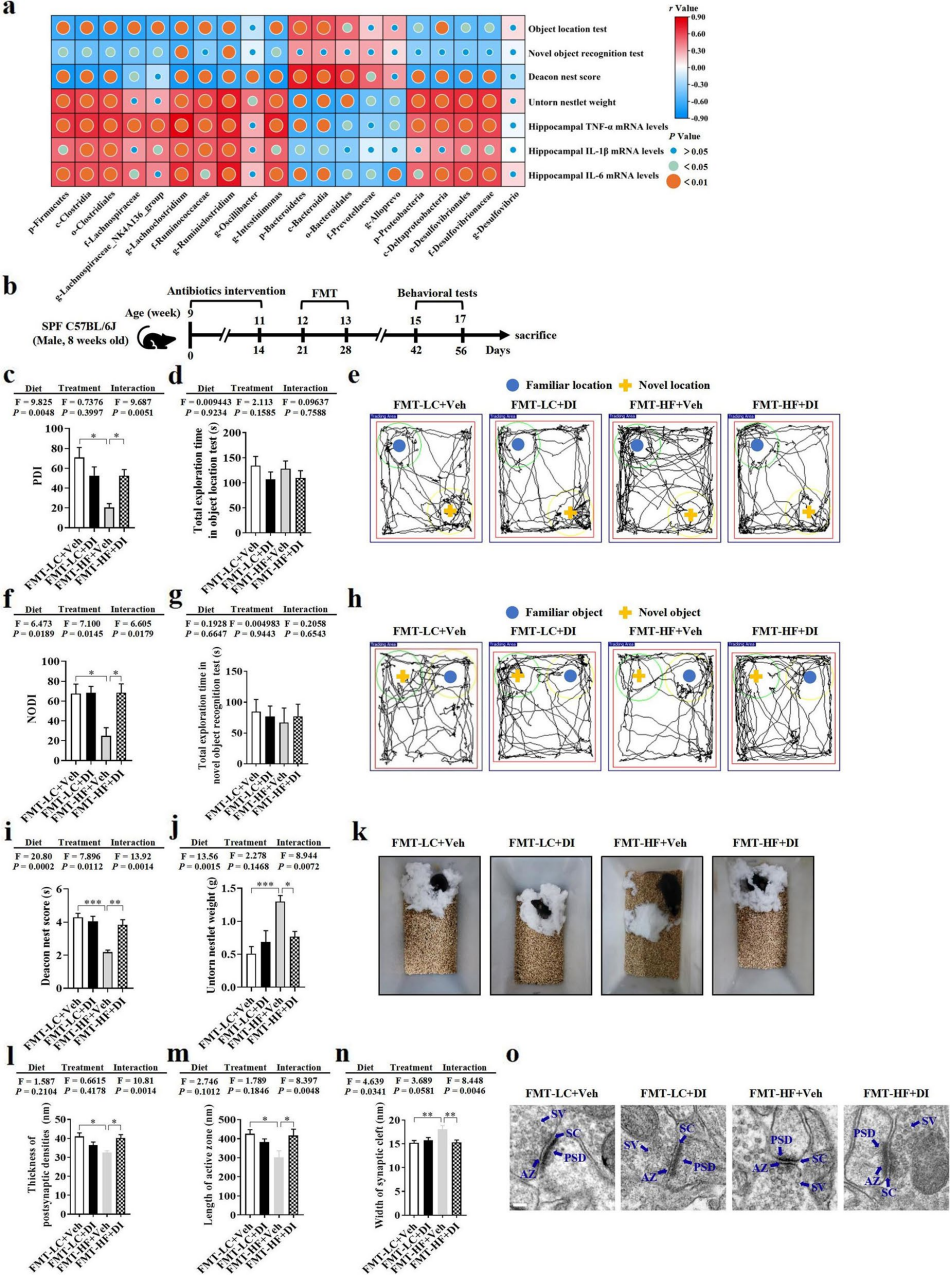
DI supplementation improved cognition and synaptic ultrastructure through the gut microbiome. **a** Pearson’s correlations among the effects of DI supplementation on gut microbiome, neuroinflammation of the hippocampus, and cognitive behaviors. **b** Schematic strategy for fecal microbiome transplantation. **c** Percentage of time spent with the object in the novel place to total object exploration time. **d** The total object exploration time in the object location test. **e** Representative track plots in object location test. **f** Percentage of time spent with the novel object to total object exploration time. **g** The total object exploration time in novel object recognition test. **h** Representative track plots in the novel object recognition test. **i** The nest score and **j** untorn nestlet weight. **k** Representative nest results in nesting building tests. *n* = 8 mice for each group in these behavior tests. **l**–**n** Statistical analysis of the thickness of PSD, length of AZ, and width of SC in the CA1 region of the hippocampus (*n* = 2, 8 images per mouse). **o** Representative ultrastructure of synapses in the CA1 region of mouse hippocampus on the electron micrograph (scale bar: 100 nm). Values are mean ± SEM. ^*^P < 0.05, ^**^P < 0.01, ^***^P < 0.001

Pearson’s correlation analysis showed that the abundance of Firmicutes, Bacteroidetes, Proteobacteria, and their taxonomic microbiome was significantly associated with cognitive indexes and hippocampal pro-inflammatory cytokine levels (Fig. 6a). This indicated that the altered profile of gut microbiome may play an important role in DI supplementation improving cognitive deficits and neuroinflammation induced by the HF diet. To confirm the hypothesis, we orally delivered the microbiome from the LC+Veh, LC+DI, HF+Veh, and HF+DI groups mice to antibiotic-pretreated C57BL/6J mice (Fig. 6b). Analysis of the fecal samples from all recipient mice demonstrated that the adoptive transfer protocol successfully produced core microbiomes in the four groups of mice. In brief, similar to the microbiota pattern of donor mice, the recipient mice receiving the microbiome from the HF diet with DI supplementation had an increase in the Shannon and Chao1 indexes, a decrease in the abundance of Firmicutes and Proteobacteria phylum, and the increase in the abundance of Desulfobacterota phylum. These mice increased the abundance of butyrate-producing bacteria compared with the recipient mice receiving the microbiome from HF diet-fed mice (Fig. S5a-h).

Importantly, microbiome from HFD-fed mice induced hippocampal-dependent cognitive impairment, evidenced by that mice transplanted with microbiome from the HF+Veh group showed lower cognitive index, PDI in object location test (*P* < 0.05, Fig. 6c, e), NODI in novel object recognition test (*P* < 0.05, Fig. 6f, h), and the nesting building ability in nest building test (both *P* < 0.05, Fig. 6i–k), compared to the mice transplanted with microbiome from the LC+Veh group. These results indicate that gut microbiome is essential in HF diet-induced hippocampus-dependent cognitive impairment. However, mice transplanted with microbiome from the HF+DI group showed higher cognitive indexes in behavior tests (all *P* < 0.05, Fig. 6c–k). Furthermore, mice transplanted with microbiome from LC+Veh and HF+DI groups exhibited thicker postsynaptic densities, longer active zone, and narrower synaptic cleft than those indexes in the mice received the microbiome from HF+Veh mice (all *P* < 0.05, Fig. 6l–o). Taken together, these results demonstrated that DI ameliorated cognitive impairment via reshaping the composition of gut microbiome.

## Discussion

**Fig. 7:**
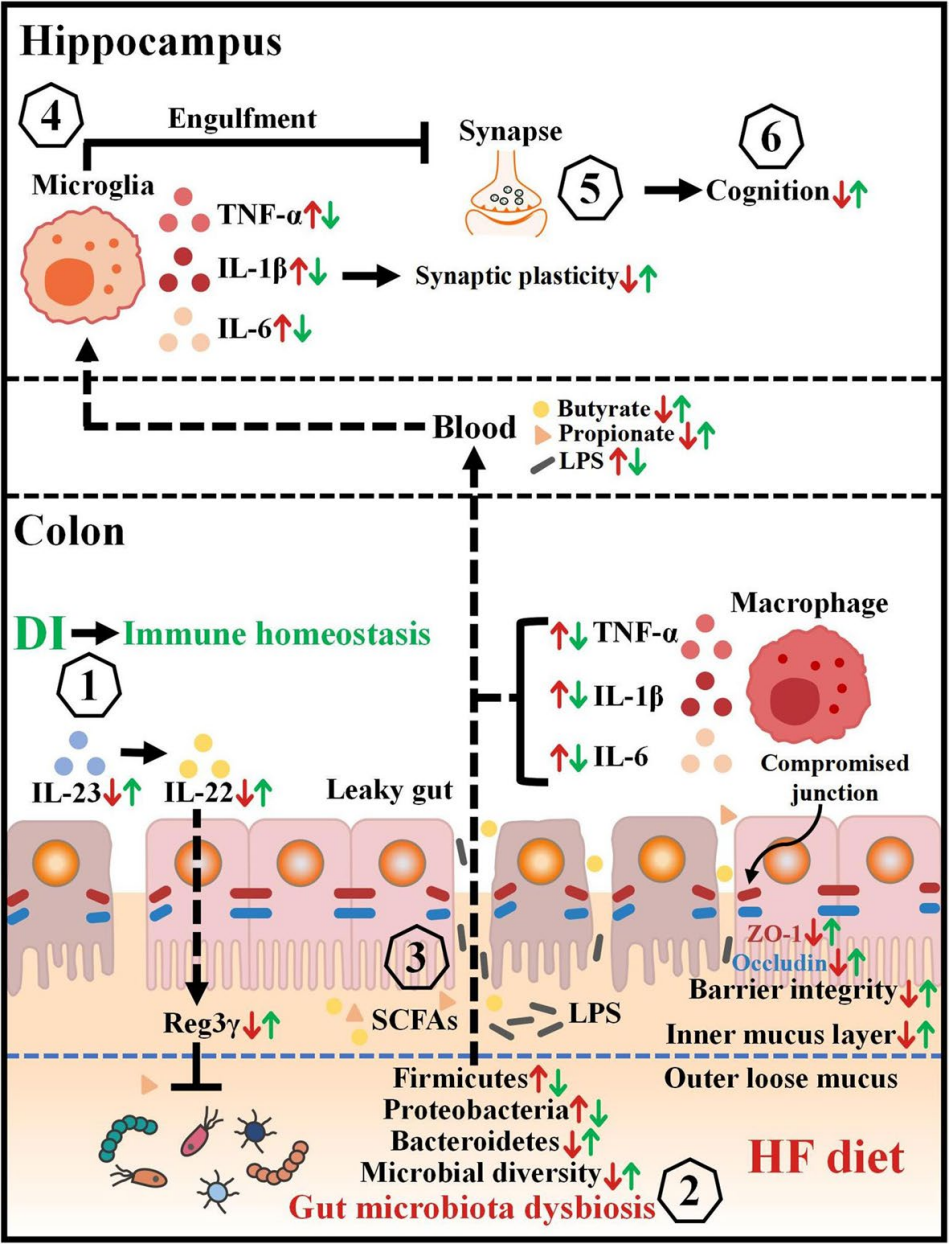
Schematic strategy for DI’s role in improving cognitive impairment induced by HF diet via the gut-brain axis. DI supplementation is proposed to alleviate the macrophage infiltration, upregulate the expression of IL-23, IL-22, and Reg3γ in the colon (1), which jointly prevent HF diet-induced microbiome shift (2). Then, the rescued gut microbiome can produce more butyrate and propionate to repair the compromised intestinal integrity (3). This decreases the levels of pro-inflammatory mediators in the blood and the brain, thereby mitigating neuroinflammation (4) and synaptic damage in the hippocampus (5), which ultimately improves cognition (6). Therefore, the supplementation of DI has a beneficial impact on cognition via the gut-brain axis. Red arrows represent altered indices induced by the HF diet, while green arrows show the protective effects of DI in HF diet-fed mice

In the present study, we reported a range of beneficial effects of DI supplementation on the gut-brain axis by using a HF diet-induced cognitive deficit model (Fig. 7). We showed that DI supplementation ameliorates cognitive deficits, as well as improves synaptic ultrastructure and neuroinflammation in the hippocampus of HF diet-fed mice. Moreover, we reported that DI supplementation effectively restores colonic immune dyshomeostasis and alleviates the impairment of the colonic mucosa barrier in the colon of mice on the HF diet. In addition, we showed that DI supplementation improves the shift of gut microbiome in HF diet-fed mice, which is characterized by the abundance increase of propionate- and butyrate-producing bacteria. Interestingly, the FMT experiment confirmed the vital role of gut microbiome in DI’s pro-cognitive effect. Collectively, these data strongly support DI as a novel drug candidate for treating HF diet-induced cognitive impairment via the gut-brain axis (Fig. 7).

Long-term intake of a HF diet can induce gut immune dysfunction, which leads to the integrity impairment of gut barrier and systemic inflammation [5, 33, 34]. We found that DI effectively reduced macrophage infiltration and downregulated mRNA levels of pro-inflammatory cytokines in the colon of mice on the HF diet. Interestingly, we found that DI supplementation upregulated the expression of immune homeostasis-related cytokines (IL-22, IL-23). It is reported that IL-23/IL-22 cytokine network is instrumental for antibacterial peptide production and host defense following intestinal damage [29, 35]. Studies have reported intestinal epithelial cells expressing IL-23 [30, 36, 37]. Here, we found that the decreased IL-23 partially recovered in the colonic epithelium of HF mice after DI administration. It should be pointed out that IL-23 can also be produced by immune cells (e.g., macrophages and dendritic cells). Therefore, future studies will investigate if IL-23 increases in intestinal immune cells after DI administration. Furthermore, IL-23 can promote Th17 to produce cytokines such as IL-22 [28]. It is reported that IL-22 can reduce microbiome encroachment by fortifying the epithelium barrier through increasing the expression of Reg3γ [29]. Consistently, we found that the mRNA expression of Reg3γ in the colon was elevated in the HF diet-fed mice after DI supplementation. Furthermore, the impairment of the gut barrier induced by the HF diet was attenuated after DI supplementation. In detail, DI supplementation effectively prevented HF diet-induced degradation of the colonic mucosal barrier, characterized by a thicker mucus layer and increased expression of tight junction proteins (ZO-1 and occludin). Correspondingly, serum LPS level was significantly decreased in HF diet-fed mice after DI supplementation, resulting from the reduced permeability due to the restoration of gut barrier integrity. Overall, DI supplementation can maintain gut immune homeostasis and alleviate HF diet-induced gut barrier impairment.

The intestinal immunity interacting with microbiome is essential for gut homeostasis [11]. The aforementioned IL-23/IL-22-Reg3γ axis not only protects the integrity of the intestinal barrier, but also inhibits the expansion of pathogenic bacteria, thereby regulating gut microbiome component [11, 31]. In the present study, the abundances of the phyla, Firmicutes, and Bacteroidetes under obese conditions were rescued after DI supplementation. Bacteroidetes are beneficial to their host intestinal mucosa and barrier function [38]. Furthermore, DI supplementation decreased the abundance of the family Ruminococcaceae, a taxon of Firmicutes, which is known to contribute to mucus layer degradation [39]. Moreover, we also found that DI supplementation decreased the abundance of Proteobacteria, a major source of antigen LPS [40]. In agreement, the abundance of predicted KEGG pathway “lipopolysaccharide biosynthesis” was decreased. To our attention, DI supplementation increased the abundance of butyrate-producing genera in HF diet-fed mice. *Lactobacillus*, contributing to the production of butyrate [41], was also increased in the present study. Propionate is mainly produced from succinate by Bacteroidetes [32]. The increase of the abovementioned bacteria may collectively contribute to the increase of butyrate and propionate in the serum of HF diet-fed mice after DI supplementation. It is reported that butyrate and propionate are energy sources of intestinal epithelial cells and the key metabolites to reduce inflammation and improve intestinal barrier integrity in the gut [42]. Therefore, DI-induced restoration of gut immune balance may ameliorate gut microbiome disorder, thereby maintaining gut homeostasis.

Gut microbiome alteration is closely implicated in cognitive decline induced by obesity via the gut-brain axis [5, 43–45]. For instance, the transplantation of obese-type microbiome impairs gut barrier and cognition in mice [9]. In the present study, DI supplementation significantly increased the α-diversity (chao1 and Shannon indexes), which was decreased by the HF diet. It has been reported that the diversity of fecal microbiome is decreased in AD patients compared to cognitively healthy controls in a prospective and cross-sectional study [46]. A previous cohort study showed that microbiome belonging to Bacteroidetes are associated with cognition and neurodegenerative diseases in infants at 1 to 2 years old [47]. Moreover, infants with high levels of gut *Bacteroides* and *Lactobacillus* at 1 year of age show higher cognitive ability at 2 years of age [48]. The proportion of Proteobacteria is increased in individuals with mild cognitive decline in clinical studies [49]. Recently, our study showed that supplementing with SCFAs (acetate, butyrate and propionate) can rescue the abnormal cognitive behavior of the fiber-deprived diet-induced obese mice [39]. Moreover, gut microbiota-derived propionate has been shown to mediate the neuroprotective effect in the mouse model of Parkinson’s disease [32]. In addition, PICRUSt predicted that in comparison with the HF+Veh group, the relative abundance of gut microbiome associated with “Alzheimer’s disease” was dramatically decreased in the HF+DI group. Therefore, DI supplementation in preventing gut microbiome alterations might contribute to improving cognitive impairment induced by HF diet.

The present study showed that the HF diet significantly decreased the cognitive indexes in the object location test, novel object recognition test, and nesting building test, compared with the LC diet. Notably, the HF diet for 15 weeks decreased the PDI from almost 70 to 20% and NODI from 70 to 30% in object location and novel object recognition tests. Previously, it is reported that both the PDI and NODI were decreased from 70 to 35% in mice after a Western-style diet (high-fat and fiber deficiency) for 11 weeks [5]. Moreover, Fu et al. found that rats fed on a HF diet for 24 weeks exhibited a remarkably reduced time spent with the novel object (~50%) [50]. Collectively, these results suggest that long-term HF diet produces may impair cognitive function. Interestingly, DI supplementation effectively improved cognitive deficits induced by the HF diet, indicating enhanced recognition memory, spatial memory, and ability to perform activities of daily living [5, 43, 44]. Furthermore, RNA-seq analysis of hippocampal tissue uncovered a sea of upregulated DEGs enriched in the GO terms associated with behavior and cognition. In addition, KEGG pathways associated with synapse and axon were also significantly enriched, including glutamatergic synapses, cholinergic synapses, and dopamine synapses. Accumulating evidence suggests that the dysfunction of glutamatergic synapses is a common pathogenic mechanism underlying neurodevelopmental disorders [51]. Moreover, cholinergic and dopaminergic neurotransmission is important for hippocampal-dependent synaptic plasticity and memory regulation [52, 53]. Therefore, DI installing altered hippocampal transcriptome may improve HF diet-induced cognitive decline.

Impairment of synaptic ultrastructure and plasticity in the hippocampus has been closely implicated in obesity-induced cognitive decline [5, 43, 44] and AD patients [54–56]. Using transmission electron microscopy, we observed thinner postsynaptic densities, shorter active zone, and wider cleft of synapse in HF diet-fed mice, while DI supplementation significantly alleviated such impairments. Synaptic associated proteins, including BDNF, SYN, and PSD95, are key mediators of synaptic plasticity and synaptogenesis [5, 57]. Reduction in BDNF, SYN, and PSD95 has been reported in the hippocampus of patients with AD or cognitive decline [54–56]. Here, the protein levels of hippocampal BDNF, SYN, and PSD95 were significantly lower in HF diet-fed mice, while DI supplementation effectively prevented the decreased expression of these synaptic proteins. Thus, it is speculated that DI supplementation can significantly ameliorate the damage to synaptic ultrastructure and the deficit of synaptic proteins induced by the HF diet. Furthermore, activated microglia can over-engulf synapses, thus mediating synapse loss and damaging synaptic ultrastructure, and consequently, cognitive dysfunction [3, 27, 58, 59]. In the present study, the activation of microglia induced by the HF diet was alleviated after DI supplementation, evidenced by the decreased microglial number and decreased mRNA levels of CD68 (the maker of activated microglia) in the hippocampus. Moreover, DI supplementation downregulated the mRNA expression of pro-inflammatory cytokines in the hippocampus of HF diet-fed mice. Our results are supported by a recent study that DI suppresses microglia activation and ameliorates neuroinflammation in a mouse model of multiple sclerosis [22]. Therefore, DI supplementation could attenuate microglial activation and neuroinflammation, consequently preventing synaptic ultrastructure impairment in HF diet-fed mice.

Gut microbiome is a key regulator of neural function via the gut-brain axis. In the present study, we showed that almost the same effects on hippocampal-dependent cognitive impairment were induced by the gut microbiome from HF diet-fed mice into the recipient mice, highlighting the critical role of the gut microbiome in mediating cognitive impairment induced by HF diet. Fecal microbiome transplantation can induce poor behavioral performance in the recipient mice. For example, microbiota transplantation from obese human subjects leads to decreased memory scores in recipient mice’s novel object recognition test [60]. The mice given the microbiota of obese donor mice induced by the HF diet have significantly decreased memory in the marble-burying behavior test without increased body weight [9]. In addition, the fecal microbiome from 5xFAD mice into normal C57BL/6 mice (5xFAD-FMT) decreases adult hippocampal neurogenesis and memory impairment in the Morris water maze test [61]. These findings, including ours, indicate that an altered gut microbiome may impair cognition, and targeting the gut microbiota may be a useful therapeutic strategy for developing novel candidates for treating cognitive deficits in obesity.

Importantly, we demonstrated that DI-induced gut microbiome shift might be the essential starting point for improving cognition by FMT experiment. Here, we reported that DI-induced improvement of behavioral phenotype and synaptic ultrastructure was transferred to C57BL/6J mice pretreated by antibiotics. Thus, this finding supports that gut microbiome mediates DI’s pro-cognitive effects. It is worth noting that the effects of DI on obesity and its related metabolic disorder in mice were not parallel to its improvement on cognition. It is reported that microbiome derived from obese mice can induce neurobehavioral changes in the absence of obesity [9]. This further explains that DI can rescue HF diet-induced cognitive deficits via regulating gut microbiome, but not affect the body weight and metabolic parameter. Overall, the present study identified the key role of gut microbiome in the DI’s protective effect on cognition.

Furthermore, in the behavioral tests, if the single detrimental factor has caused serious damage to cognitive function, multiple detrimental factors may not cause even lower cognitive indexes than the single detrimental factor. Although the present study observed the same effects on altered cognition induced by microbiome of HF diet-fed mice, we cannot conclude that gut microbiome is the only factor mediating HF diet-induced cognitive alteration. A study showed that a long-term HF diet can increase the permeability of the blood-brain barrier contributing to cognitive deficits [62]. Also, studies have reported that HF diet-induced hyperinsulinemia can impair the synaptic plasticity, neuronal survival, learning, and memory by affecting the insulin signaling in the central nervous system [63]. Therefore, it should be noted that the multiple factors are involved in obesity-associated cognitive impairments; however, gut microbiome is one of the vital factors in the HF diet-induced cognitive decline.

## Conclusion

The present study reported that DI supplementation has a protective effect against HF diet-induced cognitive impairment via optimizing synapse and cognition associated genes and alleviating neuroinflammation in the hippocampus. Furthermore, DI supplementation restores colonic immune homeostasis, ameliorates leakage of the gut barrier, and reshapes the gut microbiome profile in HF diet-fed mice. Notably, the fecal microbiome transplantation experiment uncovered that DI’s effect on cognition is dependent on gut microbiome. Overall, these findings demonstrated for the first time that DI can ameliorate HF diet-induced cognitive deficits via the gut-brain axis, which provides a novel insight for treating obesity-associated neurodegenerative diseases.

## Methods

### Animals

C57BL/6J male mice (8 weeks old) were obtained from the Experimental Animal Center of Xuzhou Medical University and housed in environmentally controlled conditions (temperature 22 °C, 12h light/dark cycle). After acclimatization to the laboratory conditions for 1 week, the mice were used for experiments approved by the Institutional Animal Care Committee of Xuzhou Medical University following the Chinese Council on Animal Care Guidelines.

### Study design and experimental timeline

#### The effect of DI administration on HF diet-induced cognitive impairment

C57BL/6J mice were randomly divided into 4 groups (*n* = 12 per group): (1) mice fed a lab chow (LC) diet (5% fat by weight) and intraperitoneally injected with phosphate buffered saline (PBS) twice a week as the LC+Veh group. (2) The LC + DI group mice had lab chow diet and intraperitoneally injected with 25 mg/kg DI (Sigma-Aldrich, Cat. 617527, St. Louis, USA) twice a week; (3) mice receiving the HF diet (30% fat by weight) and intraperitoneally injected with PBS twice a week as the HF+Veh group; (4) mice receiving the HF diet and intraperitoneally injected with DI (25 mg/kg) twice a week as the HF+DI group. The LC diet (lab autoclavable rodent diet for maintenance, Beijing Keao Xieli Feed Co., LTD) contains 5% fat, 19.6% protein, and 59.3% carbohydrate by weight. This diet is grain-based formulation, including ground corn, dehulled soybean meal, fish powder, ground wheat, brewers dried yeast, soybean oil, salt, multivitamin, and minerals. The HF diet (31.5% fat, 18.3% protein, and 38.5% carbohydrate by weight) was made from semi-synthetic materials according to the recommendation of “AIN93 Diet for Laboratory Rodents”. The detailed composition is lard 260 g, soybean oil 55 g, cornstarch 193 g, sucrose 192 g, gelatine 50 g, casein 130 g, methionine 3 g, cellulose 51 g, minerals 50 g, and vitamins 13 g.

All the groups were given intervention for 17 weeks. The body weight and food intake of mice were measured on the last day of each week. Mice were sacrificed 3 days after behavioral testing with CO_2_. Liver and fat pads (subcutaneous, epididymal, and brown) were dissected and weighed. Colon and brain tissues were immediately collected for further analyses.

#### Fecal microbiome transplantation experiment

All the mice receiving fecal microbiome transplantation (FMT) were fed on lab chow diet (lab autoclavable rodent diet for maintenance, Beijing Keao Xieli Feed Co., LTD). The mice were randomly divided into 4 groups (*n* = 8 per group): (1) FMT-LC+Veh group; (2) FMT-LC+DI group; (3) FMT-HF+Veh group; (4) FMT-HF+DI group. Firstly, mice received a combination of broad-spectrum antibiotics in their drinking water containing neomycin (1 g/L), ampicillin (1 g/L), metronidazole (1 g/L), and vancomycin (0.25 g/L), which was renewed three times per week for successive 2 weeks in their drinking water, and then switched to normal drinking water for 7 days to eliminate the pharmacological effect of antibiotics. The protocol of the fecal microbiome transplantation experiment was modified from previous studies [64, 65] and narrated as follows. Disinfected cages were daily prepared to pool feces from the mice in the experiment “the effect of DI administration on HF diet-induced cognitive impairment”, and 100 mg feces (about 5–6 fecal pellets) were then put into new sterilized tubes before the subsequent step in the sake of maintaining the vitality of gut microbiome. Then, fecal samples were mixed with sterile PBS under anaerobic conditions at a dilution ratio of 100 mg/1000 μL. Feces were rehydrated through soaking in sterile PBS for 15 min before a thoroughgoing vibration to get homogenization. After being filtered through a pore size of 70 μm, filtering liquor was centrifuged at 4 °C for 5 min and continued to take suspension centrifugal washing to gain the final bacterial suspension, which was diluted with an equal volume of sterile PBS (approximately containing10^11^ CFU/L flora) before oral gavage to receptor mice (10 mL/kg) individually for 1 week. After the fecal microbiome was colonized in extenso for 2 weeks, the behavioral tests were determined [66]. Mice were sacrificed 3 days after behavioral testing with CO_2_. Brain tissues were immediately collected for further analyses.

### Behavioral tests

The object location, novel object recognition, and nesting behavior tests were executed to examine the effects of the HF diet and DI supplementation on recognition memory and spontaneous rodent behaviors of mice. Tests were performed with reference to previous studies [5, 43, 67–69].

#### Object location test

There were three stages in the object location test. The first stage was habituation, where the mouse was allowed to explore the open field for 5 min. After 24 h, beginning the training stage, in which the mouse was allowed to explore the arena for 5 min with two identical objects placed parallel. Finally, after 1 h, a retention session takes place. Mice were allowed to explore the arena with one of the objects remaining in the same location as in trial two and the second object moved to a new location for 5 min. The arena was cleaned with 70% ethanol to minimize olfactory cues before the commencement of trial for every rat. Testing took place in a soundproof room, in which temperature was controlled at 22–25 °C. A light source (40 W fluorescent lamp) was placed 130 cm above the arena and provides a homogeneous illumination intensity approximately at 55 lux. The place discrimination index (PDI) was calculated by using the formula: (The time spent with the object moved to a novel place/the total time spent in exploring both the object moved to a novel place and the object remaining in the familiar place) × 100.

#### Novel object recognition test

There were three stages in the novel object recognition test. The first stage was habituation, in which a mouse was allowed to explore the open field for 5 min. After 24 h, the training stage began, in which the mouse was allowed to explore the arena for 5 min with two identical objects placed parallel. After 1 h, a retention session takes place. Mice were allowed to explore the arena with one of the familiar objects, and then one novel object placed parallel for 5 min. The arena was cleaned with 70% ethanol to minimize olfactory cues before the commencement of trial for every rat. Testing took place in a soundproof room, in which temperature was controlled at 22–25 °C. A light source (40 W fluorescent lamp) was placed 130 cm above the arena and provides a homogeneous illumination intensity approximately at 55 lux. The novel object discrimination index (NODI) was calculated using the formula: The time spent with the novel object/the total object exploration time × 100.

#### Nesting behavior test

One hour before the dark phase, the mice were transferred into individual cages with wood-chip bedding. A nestlet pressed-cotton square (3.0 g) was put into each cage. The next morning, remaining nests were scored according to a previously described scoring system on a definitive 5-point nest-rating scale [66] (1: Nestlet not noticeably touched or > 90% intact; 2: Nestlet 50–90% remaining intact; 3: Nestlet 50–90 % shredded, but no identifiable nest site; 4: Nestlet > 90% shredded, flat nest within ¼ of the cage; 5: A (near) perfect nest with walls higher than the mouse body height for > 50% of its circumference). The untorn nestlet pieces were weighed. The definition of an untorn piece is more than approximately 0.1 g.

The sample sizes of behavior tests were based on the calculations using power analysis at a significance level of *α* = 0.05 (two-sided) and the power of 1 − *β* = 80%. The sample size *n* = 12 for each group was estimated based on the data of three behavior tests in the previous intervention [5, 43, 45]: mean1 = 38, mean2 = 52, SD = 8, power of 1−*β* = 0.9798 in object location test; mean1 = 0.4, mean2 = 0.6, SD = 0.1, power of 1−*β* = 0.9961 in the novel objective test; mean1 = 1.8, mean2 = 3.6, mean1 = 1.8, mean2 = 3.6, SD=0.8, power of 1−β = 0.9995 in the nesting behavior test. Calculation method is on website http://powerandsamplesize.com/Calculators/Compare-kMeans/1-Way-ANOVA-Pairwise. In the “*Fecal microbiome transplantation experiment*”, the sample size *n* = 8 each group also meet the standard mentioned above.

After the behavioral tests, mice were sacrificed and the hippocampus and colonic tissues were collected. The left hippocampus was used to measure the transcriptomic profile using RNA sequencing (*n* = 3 per group) and the protein expression of PSD95, SYN, and BDNF using western blot (*n* = 5 per group). The right hippocampus was used to measure the mRNA expression of IL-1β, IL-6, and TNF-ɑ using qPCR (*n* = 6 per group) and the synaptic ultrastructure using transmission electron microscopy (*n* = 2 per group). The remaining whole brains were collected to determine the Iba1^+^ cells by immunofluorescence staining (*n* = 3 per group). Colonic tissue was used to determine the expression of F4/80, IL-23, and ZO-1 using immunofluorescence staining (*n* = 3 per group), measure the thickness of mucus layer using Alcian blue staining (*n* = 3 per group), and detect the mRNA expression of IL-1β, IL-6, TNF-ɑ, IL-22, IL-23, and Reg3γ using qPCR (*n* = 5 per group).

### Intraperitoneal glucose tolerance test (IPGTT)

IPGTT was performed at the 15^th^ week. Mice were fasted overnight (16 h) and then intraperitoneally injected with glucose (2 g/kg body weight, Sigma-Aldrich, UK). Blood samples obtained from the tail vein at 0, 15, 30, 60, 90, and 120 min following the injection of glucose were measured with an Accu-Chek glucose meter. The curve of blood glucose over time was drawn by GraphPad Prism 8.0 software, and the total area under the curve (AUC) was calculated.

### Homeostasis model assessment (HOMA) score analysis

After the mice were fasted for 6 h, the fasting blood glucose levels of the mice were detected according to the determination method in IPGTT, and an appropriate amount of whole blood was collected by squeezing the tail vein of the mouse. Blood samples were coagulated for 1 h at room temperature and centrifuged at 12,000 rpm/min for 15 min. Subsequently, the insulin levels were determined according to the Mouse Insulin Ultra Sensitive ELISA kit (Crystal Chem, USA). The homeostasis model assessment-insulin resistance (HOMA-IR) was applied to estimate the insulin sensitivity [70] and was calculated according to the formula (HOMA-IR = blood glucose concentration × insulin concentration / 22.5).

### Transmission electron microscopy

After transcardial perfusion with saline, the left side of mice in the hippocampal CA1 region was taken and rapidly fixed in glutaraldehyde. After fixation for 24 h, the tissues were quickly dissected and separated into thin slices. They were fixed immediately with 2.5% glutaraldehyde at 4 °C overnight. Washed 3 times in PBS, these slices were fixed in 1% osmium tetroxide, stained with 2% aqueous solution of uranyl acetate, and then dehydrated with different concentrations of ethanol and acetone gradient. Finally, they were embedded in epoxy resin. Sections (70 nm) were cut and stained with 4% uranyl acetate and lead citrate. Synapses can be classified into asymmetric and symmetric synapses, or Gray I type and Gray II type synapses, which are considered to mediate excitatory and inhibitory transmission, respectively. Asymmetric synapses have prominent postsynaptic densities and relatively wide synaptic clefts, while symmetric synapses are with pre- and postsynaptic densities of equal thickness and narrower synaptic clefts. Asymmetric synapses were specially examined for excitatory synaptic measurement in the present study. PSD thickness, SC width, and AZ length were determined using ImageJ software as described previously [43].

### Immunofluorescence staining

Mice were transcardially well-perfused with 0.9% saline infusion followed by 4% paraformaldehyde in 0.1 M phosphate buffer. The tissues of the colons and the brains were dissected and immediately placed in 4% paraformaldehyde for 24 h, and next in 4% paraformaldehyde containing 30% sucrose. The brain and the colon tissues were embedded in paraffin and sliced into 3-μm-thick and 5-μm-thick slides, respectively. Briefly, the slides went through antigen retrieval using sodium citrate solution (pH 6.0). After being blocked with 3% bovine serum albumin (Servicebio, G5001), the slides were incubated with primary antibodies at 4 °C overnight. The primary antibodies include the following: anti-F4/80 (Servicebio, GB113373, 1:5000 dilution), anti-ZO-1 (Servicebio, GB111981, 1:200 dilution), anti-IL-23 (Wanleibio, WL01655, 1:200 dilution), and anti-Iba-1 (Abcam, Ab178847, 1:500 dilution). Then, the appropriate secondary antibody goat anti-rabbit IgG-Cy3 (Servicebio, GB21303, 1:300 dilution) was used to detect the corresponding primary antibodies. DAPI solution (Servicebio, G1012) was used to detect nuclei. The representative images were captured by a fluorescence microscope (Nikon Eclipse C1, Tokyo, Japan). The quantification of positively stained cells was conducted using ImageJ.

### Alcian blue staining

After the methanol-stored colon samples were embedded in paraffin and cut into sections (5 μm), the alcian blue staining was performed by the protocols described previously [39]. The paraffin sections were deparaffinized in xylene and hydrated with distilled water. Then, sections were incubated with alcian blue solution for 30 min and washed in running tap water for 2 min. After being rinsed in distilled water, sections were dehydrated (2× changes) and treated with absolute alcohol (2× changes) for 3 min each. At last, they were cleared in xylene (3× changes) for 3 min each and after which the cover glass was mounted. The thickness of the colonic sections was measured using ImageJ.

### RNA extraction and quantitative (q) real-time polymerase chain reaction (qPCR)

RNA extraction and qPCR were performed based on methods previously described [43]. In short, total RNA was extracted with TRIzol (Thermo Fisher Scientific, USA) from the hippocampus and colon. RNA purity was determined by the absorption ratios (260/280 nm), which were 1.8–2.0 for all samples. One microgram purified RNA was used to generate cDNA with a High-Capacity cDNA Reverse Transcription Kit (Takara, Dalian, China) and qPCR was performed using the SYBR GREEN Master Mix (TaKaRa, Japan) and determined on a real-time PCR detection system (Bio-Rad, USA). The CT value of each reaction was provided, and the changes in transcriptional level of the target gene normalized to β-actin were calculated by the following formula: Relative mRNA level of target gene (folds of control) = 2^−ΔΔCT^. Primer sequences were supplemented in Table S11.

### Western blotting

Western blot assay was performed as described previously [43]. Briefly, the mouse hippocampus was homogenized in ice-cold RIPA lysis buffer, supplemented with a complete EDTA-free protease inhibitor cocktail and PhosSTOP Phosphatase Inhibitor. The supernatant was collected, and a BCA assay was used to quantify the protein concentration. Equal amounts of protein were separated by sodium dodecyl sulfate-polyacrylamide gel electrophoresis (SDS-PAGE) and transferred onto polyvinylidene difluoride (PVDF) membranes. The membrane was blocked with 5% non-fat milk at room temperature for 1 h and then incubated overnight at 4 °C with different primary antibodies: anti-SYN (Abcam, ab32127, 1:50,000 dilution), anti-PSD95 (Cell Signaling Technology, #3450, 1:1000 dilution), anti-BDNF (Abomone labs, ANT-010, 1:200 dilution), and β-actin (ABclonal, AC026, 1:50,000). The membranes were washed 3 times for 10 min and incubated with HRP-inked anti-rabbit IgG secondary antibody (Cell Signaling Technology, 7074, 1:5000 dilution) or HRP-linked anti-mouse IgG secondary antibody (Cell Signaling Technology, 7076S, 1:5000 dilution) at room temperature for 1 h. After washing 3 times with TBST, the protein bands were detected with Clarity™ ECL western blot substrate (Bio-Rad, 1,705,060) and visualized using the ChemiDoc Touch imaging system (Bio-Rad). The expression of protein in each sample was normalized to β-actin.

### 16S rRNA gene sequencing

Fecal samples were collected from the mice in the experiment of “*The effect of DI administration on HF diet-induced cognitive impairment*” upon defecation in the late afternoon at the 15th week and were stored at −80°C. The genomic DNA was extracted using the E.Z.N.A. stool DNA Kit (Omega Bio-tek, Norcross, GA, USA) according to the manufacturer’s protocols. V3–V4 variable regions of 16S rRNA genes were amplified by PCR (94 °C for 5 min, followed by 26 cycles at 94 °C for 30 s, 56 °C for 30 s, and 72 °C for 20 s; and a final extension at 72 °C for 5 min) with universal primers 343 F: TACGGRAGGCAGCAG; 798 R: AGGGTATCTAATCCT. Amplicon quality was visualized using gel electrophoresis, purified with AMPure XP beads (Agencourt), and amplified for another round of PCR. After purifying with the AMPure XP beads again, the final amplicon was quantified using the Qubit dsDNA assay kit. Purified amplicons were pooled in equivalent molar and paired-end sequences (2 × 250) on an Illumina platform according to the standard protocols. Sequences with ≥ 97% similarity were assigned to the same operational taxonomic units (OTUs). LEfSe was performed to detect the features of gut microbiome with significant difference abundances between designated taxa with the Kruskal−Wallis rank sum test. Based on the KEGG functional pathway, the predicted functional composition of the gut microbiome was inferred for each sample using PICRUSt. Statistical analyses were conducted with Statistical Analysis of Metagenomic Profiles (STAMP) software, and functional differences in orthologs among groups were assessed by Kruskal-Wallis tests.

### RNA sequencing

Fresh hippocampus tissues of mice were collected to analyze the whole profile of the transcriptome. Total RNA was extracted using a Trizol reagent kit (Invitrogen, Carlsbad, CA, USA) according to the manufacturer’s protocol, and mRNA was enriched by Oligo(dT) beads. The enriched mRNA was fragmented into short fragments using fragmentation buffer and reverse transcribed into cDNA. The purified double-stranded cDNA fragments were end-repaired, “A” base added, and ligated to Illumina sequencing adapters. The ligation reaction was purified with the AMPure XP Beads (1.0X). Ligated fragments were subjected to size selection by agarose gel electrophoresis and PCR amplified. The resulting cDNA library was sequenced using Illumina Novaseq6000 by Gene Denovo Biotechnology Co. (Guangzhou, China). The DESeq2 program was used to identify differentially expressed mRNAs based on their relative quantities, which were reflected by individual gene reads. Genes with a *P*-value < 0.05 and absolute fold change ≥ 1.5 were recognized as significantly differentially expressed genes between the two samples. GO enrichment analysis was performed at Metascape (https://metascape.org/gp/) [71]. Only terms with a *P* < 0.05, minimum count of 3, and enrichment factor > 1.5 were considered significant. KEGG pathway enrichment analysis was performed using the KEGG pathway database (http://www.kegg.jp/), and the pathways with *P* < 0.05 were considered significantly changed between two groups. PPI network was performed using the STRING database (https://stringdb.org/, v.10.5) and Cytoscape 3.9.1.

### Determination of short-chain fatty acid (SCFA) concentrations

SCFA levels were determined by gas chromatography-mass spectrometry (GC-MS) in serum samples freshly collected from the mice. Helium was used as the carrier gas at a constant flow rate of 1 mL/min. The initial oven temperature was held at 60 °C for 5 min, increased to 250 °C at 10 °C/min, and finally held at 250 °C for 5 min. The temperatures of the front entrance, transmission line, and electron impact (EI) ion source were set to 280, 250, and 230 °C, respectively. Data processing was performed using an Agilent MSD ChemStation (Agilent).

### Statistical analysis

Data were analyzed using GraphPad Prism software 8.0 and are presented as the mean ± standard error of mean (SEM). Two-way ANOVA was used to analyze the effects of multiple factors, including HF diet, DI treatment, and their interaction. If the main and interactive effects were defined, post hoc comparisons were performed using Tukey’s multiple comparisons test to assess the statistical significance among groups. A *P*-value < 0.05 was considered to indicate statistical significance.

## Supplementary Information

**Additional file 1: Figure S1.** DI supplementation did not reduce body weight gain and improve metabolic indices in HF diet-fed mice. a and b The body weight at 17th week and every week. c Cumulative body weight gain. d-g The mass of liver, subcutaneous fat, epididymal fat and brown fat. h The energy intake. i Fasting insulin. j Homeostasis model assessment (HOMA)-insulin resistance (IR) index. k Glycemia changes at 0,15, 30, 60, 90, and 120 min. l AUC for IPGTT. *n* = 12 mice for each group. Values are mean ± SEM. ^*^P < 0.05, ^**^P < 0.01, ^***^P < 0.001.

**Additional file 2: Figure S2.** Transcriptome analysis of GO annotation and KEGG pathways after DI supplementation. a and b Go annotation of upregulated DEGs between HF+DI and HF+Veh groups with top 30 enrichment scores covering domains of biological process (a), and cellular component (b). c The bubble chart shows the top 30 terms of KEGG pathways of DEGs between HF+DI and HF+Veh groups mice.

**Additional file 3: Figure S3.** Comparison of the taxonomic abundance of microbiota. a Comparison of the representative taxonomic abundance of Firmicutes at the class, order, family, and genus levels. b Comparison of the representative taxonomic abundance of Proteobacteria at the class, order, family and genus levels. c Comparison of the representative taxonomic abundance of Bacteroidetes at the class, order, family and genus levels. d Comparison of the representative taxonomic abundance of Bacilli at the class, order, family, and genus levels. *n* = 6 mice for each group. Values are mean ± SEM. ^*^P < 0.05, ^**^P < 0.01, ^***^P < 0.001.

**Additional file 4: Figure S4.** Predicted KEGG functional pathway differences inferred from 16S rRNA gene sequences using PICRUSt. a and b Mean abundance difference at level 1 (a) and level 2 (b). *n* = 6 mice for each group.

**Additional file 5: Figure S5.** The microbiome composition of mice receiving fecal microbiome transplantation. The microbiome composition in the fecal of the recipient mice post fecal microbiome transplantation was analyzed by 16S rRNA gene sequencing (*n* = 6-7 mice per group). a Shannon index. b Chao1 index. c Composition of abundant bacterial phyla. d-f Relative abundance of Firmicutes, Proteobacteria, and Proteobacteria. g Linear discriminant analysis (LDA) effect size (LEfSe) showing the most significantly abundant taxa enriched in microbiome from the FMT-HF+DI group compared to the FMT-HF+Veh group. h The heatmap of relative abundance of genera associated with the production of butyrate. The intensity of color in the heatmap (blue to red) indicates the normalized abundance score for each genus. Values are mean ± SEM. ^*^P < 0.05, ^**^P < 0.01. Abbreviations: p, phylum; c, class; o, order; f, family; g, genus; FMT, fecal microbiome transplantation.

**Additional file 6: Table S1.** 377 upregulated DEGs in the hippocampus between HF+Veh group mice and LC+Veh group mice. **Table S2.** 342 downregulated DEGs in the hippocampus between HF+Veh group mice and LC+Veh group mice. **Table S3.** 697 upregulated DEGs in the hippocampus between HF+DI group mice and HF+Veh group mice. **Table S4.** 185 downregulated DEGs in the hippocampus between HF+DI group mice and HF+Veh group mice. **Table S5.** Significantly enriched GO terms of upregulated DEGs between HF+DI group mice and HF+Veh group mice about biologic process. Only terms with a *P* < 0.05, minimum count of 3, and enrichment factor > 1.5 were considered significant. **Table S6.** Significantly enriched GO terms of upregulated DEGs between HF+DI group mice and HF+Veh group mice about cellular component. Only terms with a *P* < 0.05, minimum count of 3, and enrichment factor > 1.5 were considered significant. **Table S7.** KEGG pathways analysis of DEGs between HF+DI and HF+Veh groups. **Table S8.** Predicted KEGG functional pathway at level 1 inferred from 16S rRNA gene sequences using PICRUSt. **Table S9.** Predicted KEGG functional pathway at level 2 inferred from 16S rRNA gene sequences using PICRUSt. **Table S10.** Predicted KEGG functional pathway at level 3 inferred from 16S rRNA gene sequences using PICRUSt. **Table S11.** The qRT-PCR primer sequences used in this study.

## Data Availability

The RNA-seq data and 16S rRNA sequencing data in this study are available in the Sequence Read Archive (S.R.A.) under project numbers PRJNA801352 and PRJNA801432, respectively.

## Abbreviations

HF: High fat
DI: Dimethyl itaconate
AD: Alzheimer’s disease
Th17: T helper cell 17
ILC3: Type 3 innate lymphoid cells
LPS: Lipopolysaccharide
EAE: Experimental autoimmune encephalomyelitis
RNA-seq: RNA sequencing
FMT: Fecal microbiome transplantation
PDI: Place discrimination index
NODI: Novel object discrimination index
DEGs: Differentially expressed genes
GO: Gene ontology
PPI: Protein-protein interactions
CA1: Cornu ammonis 1
PSD: Postsynaptic densities
AZ: Active zone
SC: Synaptic cleft
ZO-1: Zonula occludens-1
LC: Lab chow
LDA: Linear discriminant analysis
LEfSe: Linear discriminant analysis effect size
GC-MS: Gas chromatography-mass spectrometry
SCFA: Short-chain fatty acid
PBS: Phosphate buffered saline
IPGTT: Intraperitoneal glucose tolerance test
AUC: Area under the curve
HOMA-IR: Homeostasis model assessment-insulin resistance
PCR: Polymerase chain reaction
SDS-PAGE: Sodium dodecyl sulfate-polyacrylamide gel electrophoresis
PVDF: Polyvinylidene difluoride
OTUs: Operational taxonomic units
STAMP: Statistical Analysis of Metagenomic Profiles
EI: Electron impact
SEM: Standard error of mean
ANOVA: Analysis of variance
