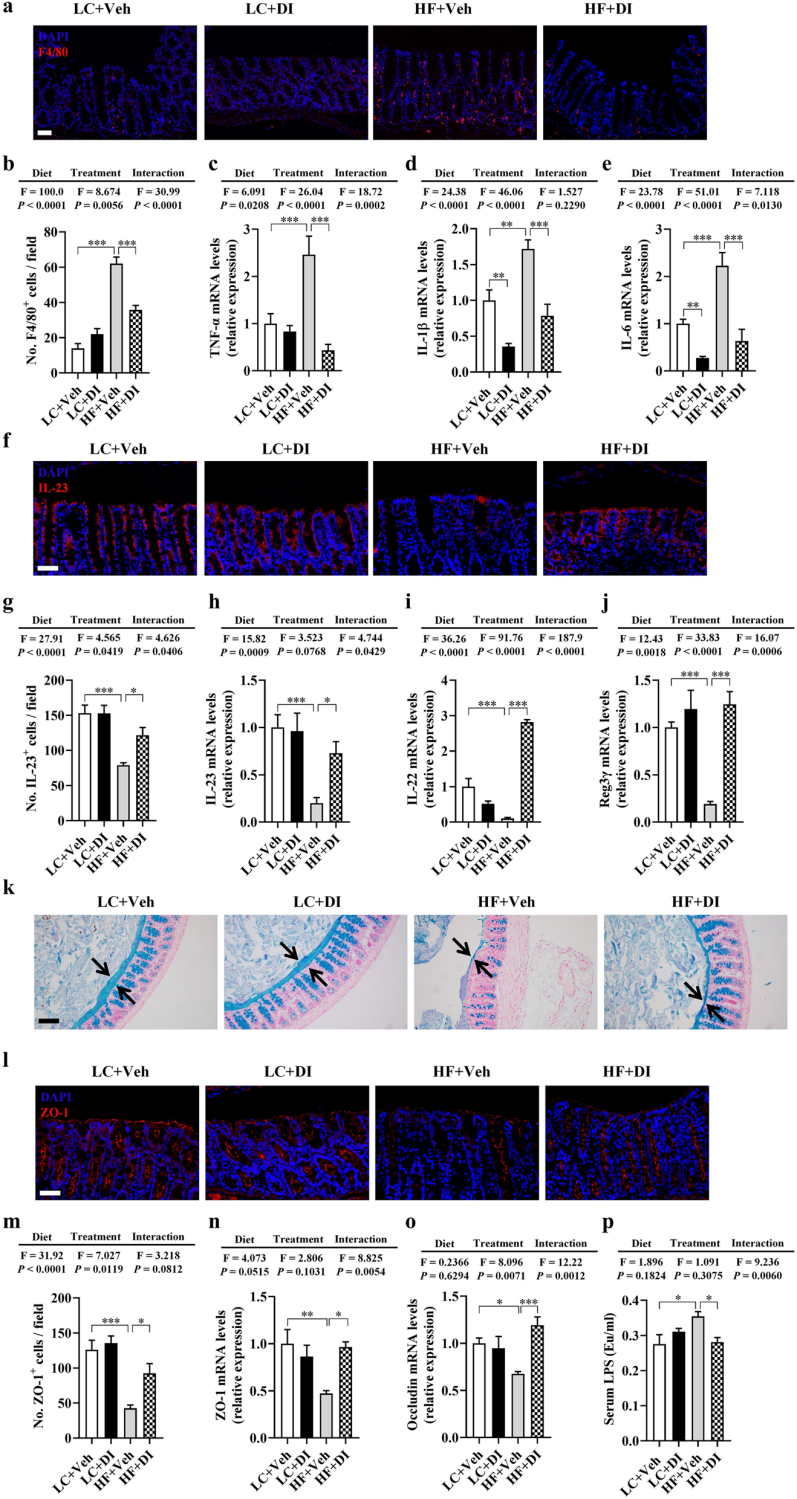# Correction: Dimethyl itaconate ameliorates cognitive impairment induced by a high-fat diet via the gut-brain axis in mice

**DOI:** 10.1186/s40168-023-01515-z

**Published:** 2023-03-20

**Authors:** Wei Pan, Jinxiu Zhao, Jiacheng Wu, Daxiang Xu, Xianran Meng, Pengfei Jiang, Hongli Shi, Xing Ge, Xiaoying Yang, Minmin Hu, Peng Zhang, Renxian Tang, Nathan Nagaratnam, Kuiyang Zheng, Xu-Feng Huang, Yinghua Yu

**Affiliations:** 1 Jiangsu Key Laboratory of Immunity and Metabolism, Jiangsu International Laboratory of Immunity and Metabolism, Department of Pathogen Biology and Immunology, Xuzhou Medical University, Xuzhou, 221004 Jiangsu China; 2 The Second School of Clinical Medicine, Xuzhou Medical University, Xuzhou, 221004 Jiangsu China; 3 Illawarra Health and Medical Research Insti- tute (IHMRI) and School of Medicine, University of Wollongong, Wollongong, NSW 2522 Australia

**Correction: Microbiome 11, 30 (2023)**

**https://doi.org/10.1186/s40168-023-01471-8**

Following the publication of the original article [1], the author reported that Fig. 4i is missing. The correct Fig. 4 is included here and the original article has been updated.