# Supplementary figures and images for: Dimethyl itaconate ameliorates cognitive impairment induced by a high-fat diet via the gut-brain axis in mice
Source: Microbiome. 2023 Feb 21;11:30. doi: 10.1186/s40168-023-01471-8 (PMC9942412; doi:10.1186/s40168-023-01471-8)

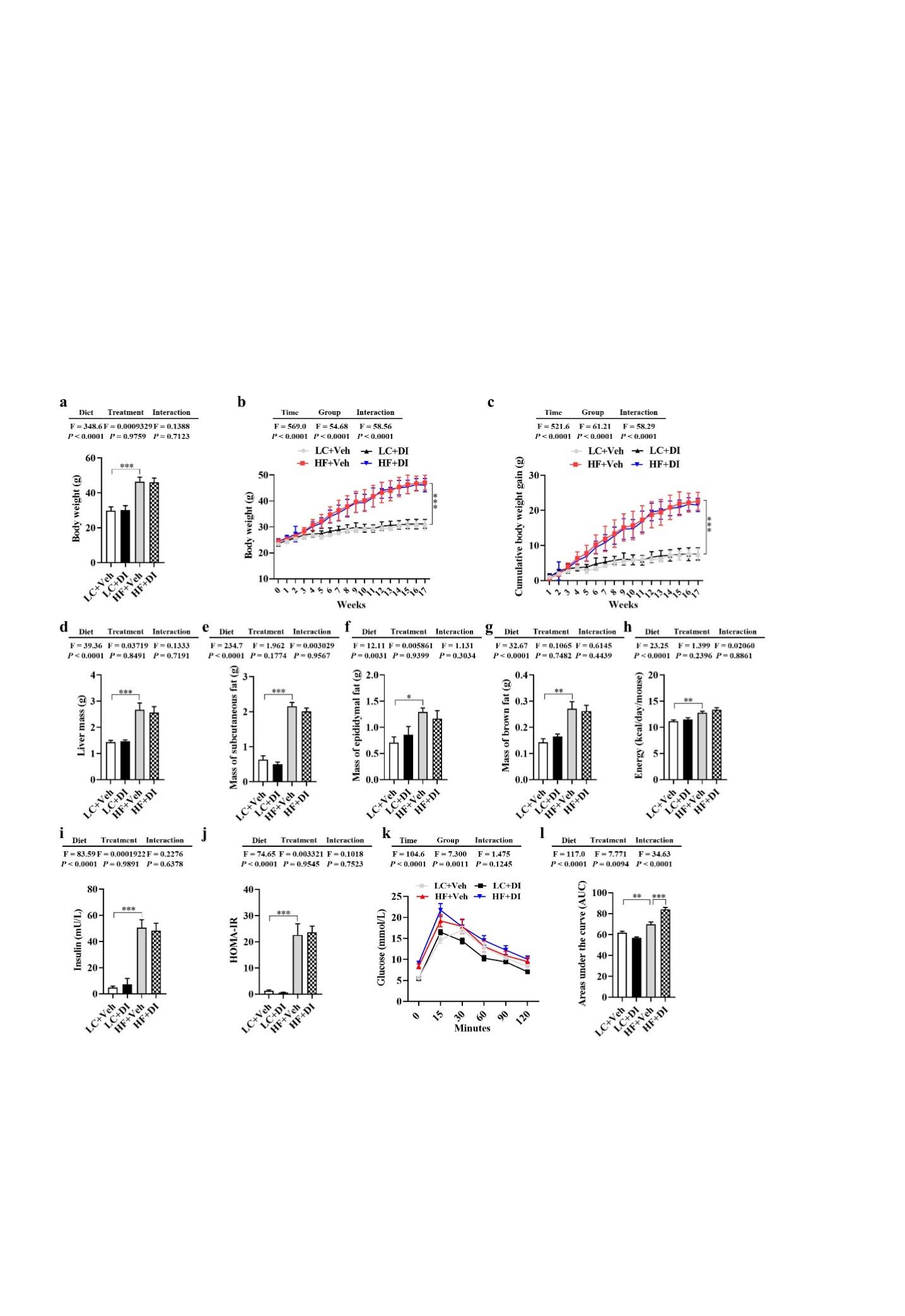

Supplement: Supplementary file 2 — Additional file 1: Figure S1. DI supplementation did not reduce body weight gain and improve metabolic indices in HF diet-fed mice. a and b The body weight at 17th week and every week. c Cumulative body weight gain. d-g The mass of liver, subcutaneous fat, epididymal fat and brown fat. h The energy intake. i Fasting insulin. j Homeostasis model assessment (HOMA)-insulin resistance (IR) index. k Glycemia changes at 0,15, 30, 60, 90, and 120 min. l AUC for IPGTT. n = 12 mice for each group. Values are mean ± SEM. *P < 0.05, **P < 0.01, ***P < 0.001. [file 40168_2023_1471_MOESM1_ESM.docx]

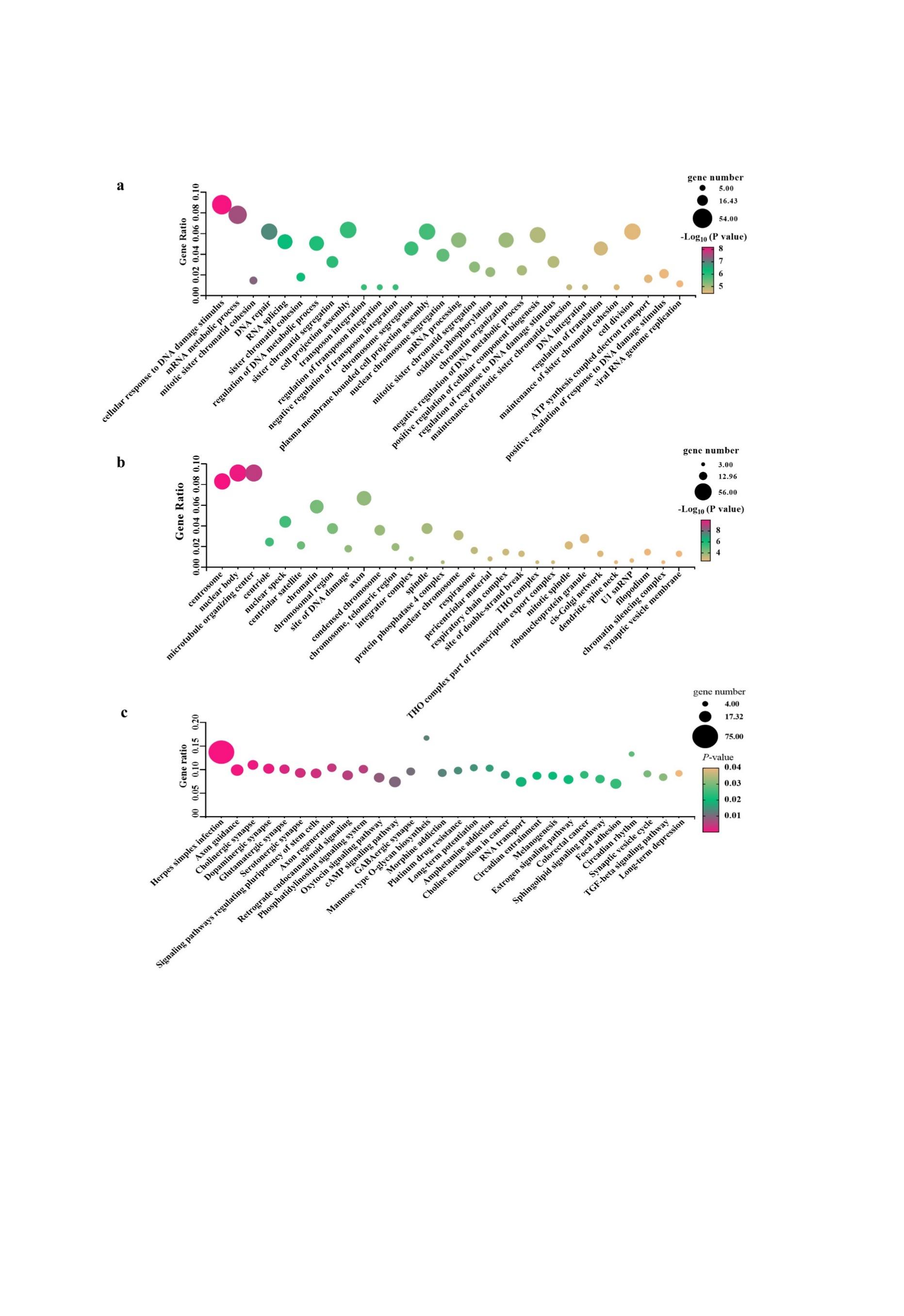

Supplement: Supplementary file 3 — Additional file 2: Figure S2. Transcriptome analysis of GO annotation and KEGG pathways after DI supplementation. a and b Go annotation of upregulated DEGs between HF+DI and HF+Veh groups with top 30 enrichment scores covering domains of biological process (a), and cellular component (b). c The bubble chart shows the top 30 terms of KEGG pathways of DEGs between HF+DI and HF+Veh groups mice. [file 40168_2023_1471_MOESM2_ESM.docx]

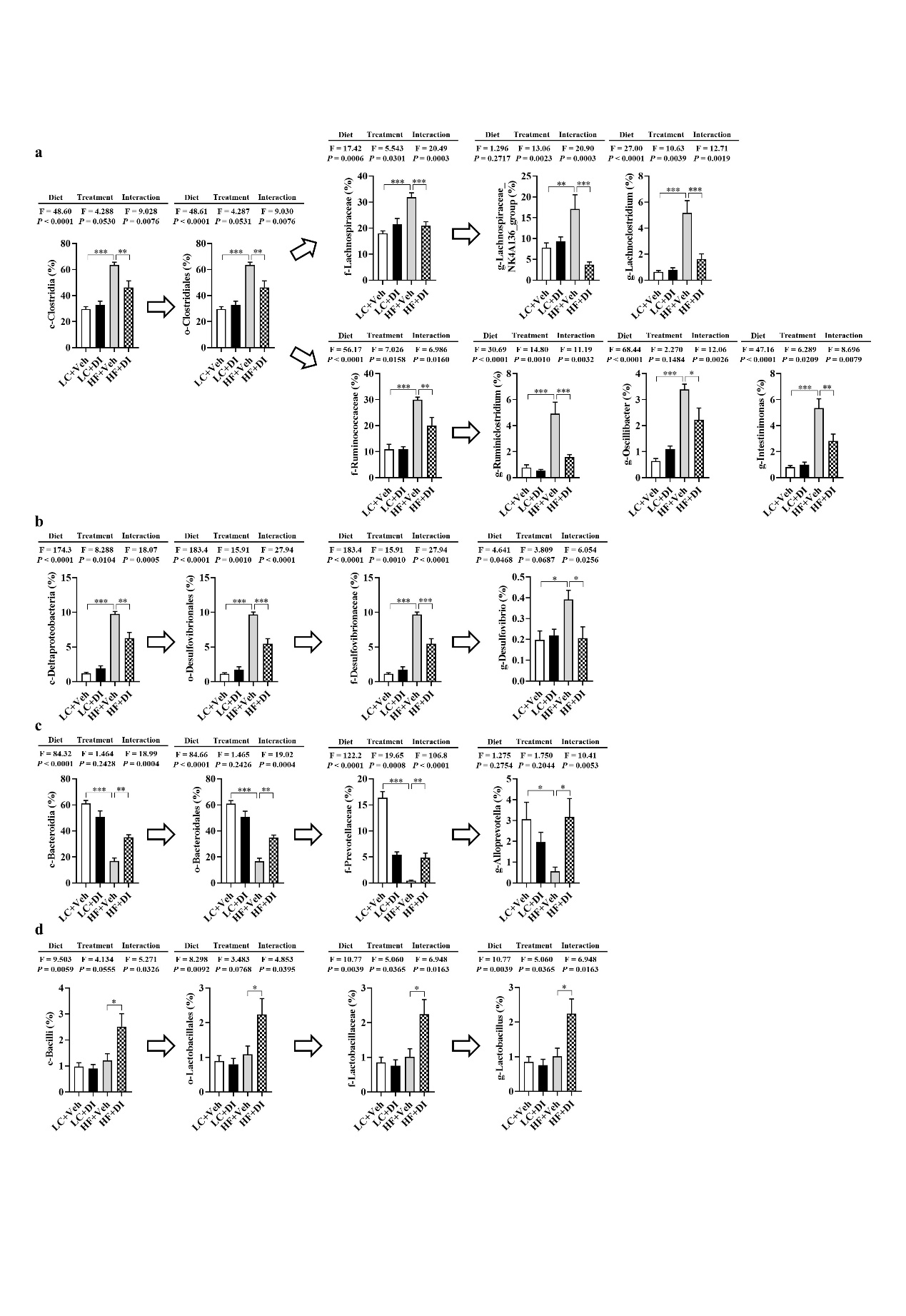

Supplement: Supplementary file 4 — Additional file 3: Figure S3. Comparison of the taxonomic abundance of microbiota. a Comparison of the representative taxonomic abundance of Firmicutes at the class, order, family, and genus levels. b Comparison of the representative taxonomic abundance of Proteobacteria at the class, order, family and genus levels. c Comparison of the representative taxonomic abundance of Bacteroidetes at the class, order, family and genus levels. d Comparison of the representative taxonomic abundance of Bacilli at the class, order, family, and genus levels. n = 6 mice for each group. Values are mean ± SEM. *P < 0.05, **P < 0.01, ***P < 0.001. [file 40168_2023_1471_MOESM3_ESM.docx]

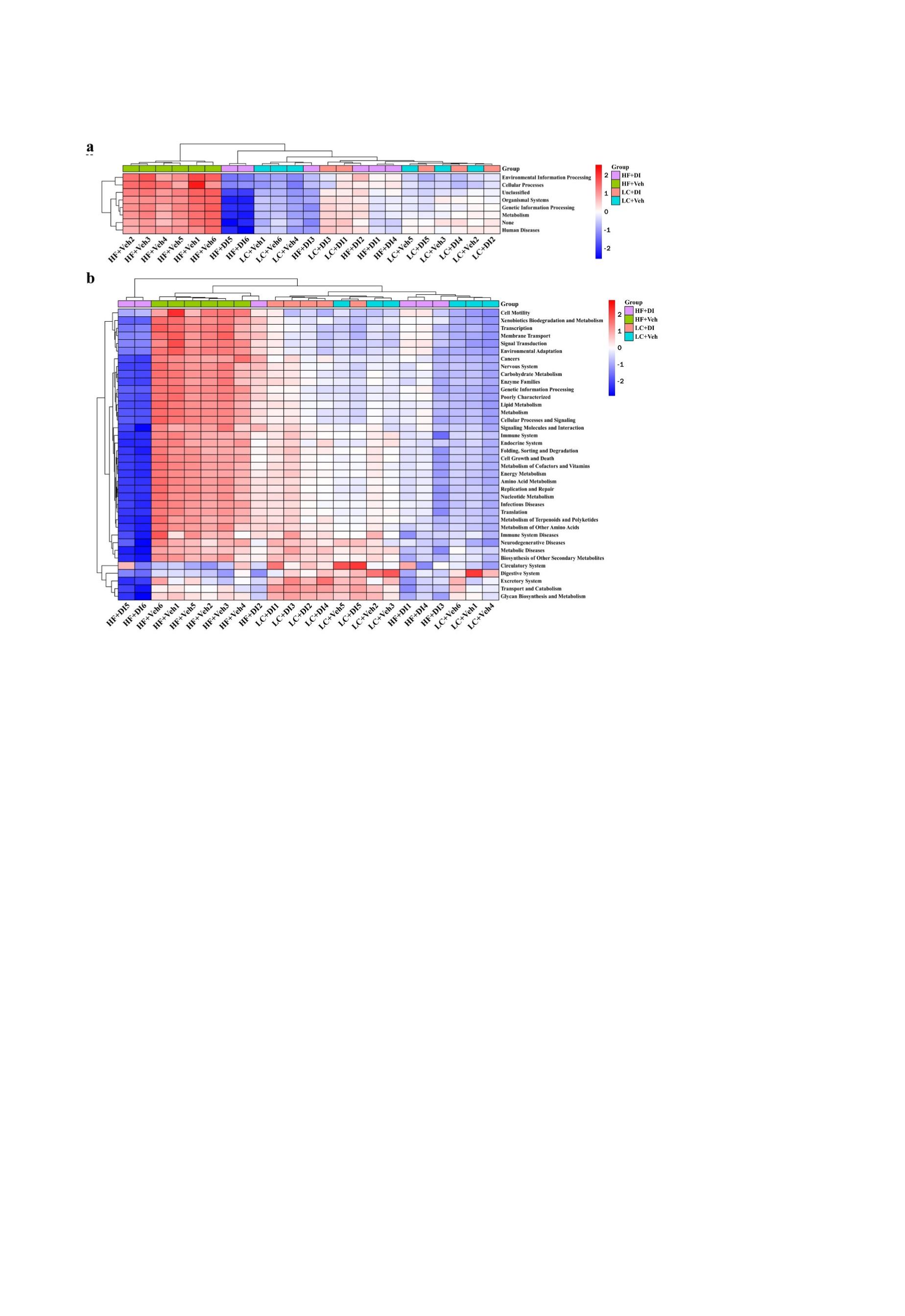

Supplement: Supplementary file 5 — Additional file 4: Figure S4. Predicted KEGG functional pathway differences inferred from 16S rRNA gene sequences using PICRUSt. a and b Mean abundance difference at level 1 (a) and level 2 (b). n = 6 mice for each group. [file 40168_2023_1471_MOESM4_ESM.docx]

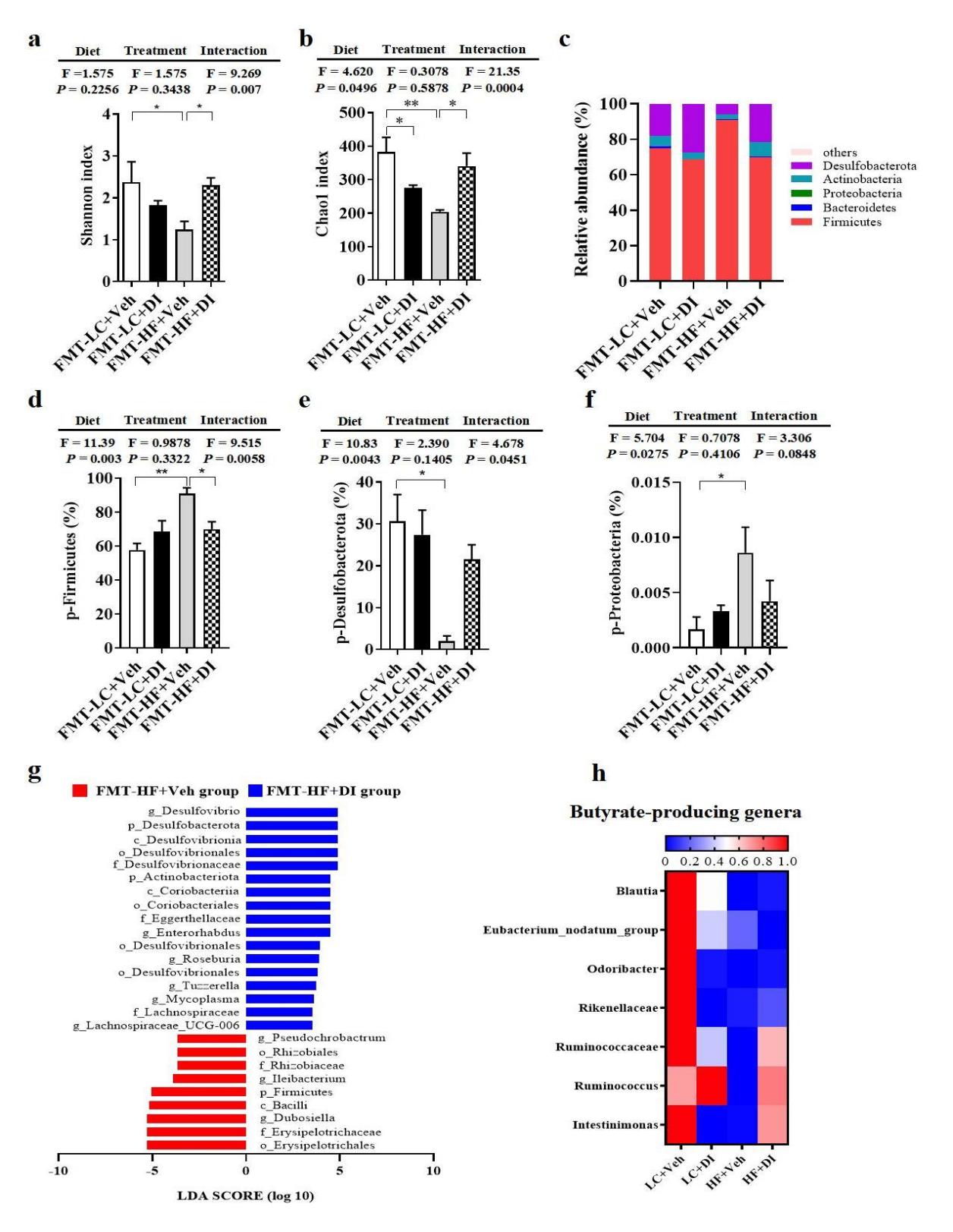

Supplement: Supplementary file 6 — Additional file 5: Figure S5. The microbiome composition of mice receiving fecal microbiome transplantation. The microbiome composition in the fecal of the recipient mice post fecal microbiome transplantation was analyzed by 16S rRNA gene sequencing (n = 6-7 mice per group). a Shannon index. b Chao1 index. c Composition of abundant bacterial phyla. d-f Relative abundance of Firmicutes, Proteobacteria, and Proteobacteria. g Linear discriminant analysis (LDA) effect size (LEfSe) showing the most significantly abundant taxa enriched in microbiome from the FMT-HF+DI group compared to the FMT-HF+Veh group. h The heatmap of relative abundance of genera associated with the production of butyrate. The intensity of color in the heatmap (blue to red) indicates the normalized abundance score for each genus. Values are mean ± SEM. *P < 0.05, **P < 0.01. Abbreviations: p, phylum; c, class; o, order; f, family; g, genus; FMT, fecal microbiome transplantation. [file 40168_2023_1471_MOESM5_ESM.docx]
